# When I Look into Your Eyes: A Survey on Computer Vision Contributions for Human Gaze Estimation and Tracking

**DOI:** 10.3390/s20133739

**Published:** 2020-07-03

**Authors:** Dario Cazzato, Marco Leo, Cosimo Distante, Holger Voos

**Affiliations:** 1 Interdisciplinary Center for Security, Reliability and Trust (SnT), University of Luxembourg, L-4364 Esch-sur-Alzette, Luxembourg; dario.cazzato@uni.lu (D.C.); holger.voos@uni.lu (H.V.); 2 National Research Council of Italy—Institute of Applied Sciences and Intelligent Systems, 73100 Lecce, Italy; cosimo.distante@cnr.it

**Keywords:** gaze tracking, gaze estimation, survey, review, computer vision

## Abstract

The automatic detection of eye positions, their temporal consistency, and their mapping into a line of sight in the real world (to find where a person is looking at) is reported in the scientific literature as gaze tracking. This has become a very hot topic in the field of computer vision during the last decades, with a surprising and continuously growing number of application fields. A very long journey has been made from the first pioneering works, and this continuous search for more accurate solutions process has been further boosted in the last decade when deep neural networks have revolutionized the whole machine learning area, and gaze tracking as well. In this arena, it is being increasingly useful to find guidance through survey/review articles collecting most relevant works and putting clear pros and cons of existing techniques, also by introducing a precise taxonomy. This kind of manuscripts allows researchers and technicians to choose the better way to move towards their application or scientific goals. In the literature, there exist holistic and specifically technological survey documents (even if not updated), but, unfortunately, there is not an overview discussing how the great advancements in computer vision have impacted gaze tracking. Thus, this work represents an attempt to fill this gap, also introducing a wider point of view that brings to a new taxonomy (extending the consolidated ones) by considering gaze tracking as a more exhaustive task that aims at estimating gaze target from different perspectives: from the eye of the beholder (first-person view), from an external camera framing the beholder’s, from a third-person view looking at the scene where the beholder is placed in, and from an external view independent from the beholder.

## 1. Introduction

Gaze is a fundamental communication mean, since it can express emotions, feelings and intentions [1]. Evidence of its importance is in the noticeable quantity of applications that have been presented in the last few decades, spacing from Human-Computer and Human-Robot Interaction (HCI/HRI) [2,3] to assistive devices [4,5,6], healthcare/clinical assessment and diagnosis [7], driver vigilance monitoring [8,9], analysis of consumer market [10], analysis of behavioral patterns in disabilities or diseases [11,12], gaming design [13,14], and so on. Gaze tracking consists of the procedure of obtaining the direction or the point of regard (PoR) of the gaze with continuity, on a digital screen or in the physical environment, and it is achieved through mechanical, electronic, optical, and/or other methods.

Although researchers often use the terms of eye tracking and gaze tracking as synonyms, there is a slight difference between them. In particular, eye tracking is the measurement of eye movement/activity, while gaze tracking is the analysis of eye tracking data with regards to the head or the visual scene (a known three-dimensional (3D) environment, a screen, a surface, etc.). In other words, eye tracking consists in detecting the existence and position of the eyes into an image and to track them over time (in the following images), and then in measuring activities as eye blinks, fixations, saccadic movements [15], pupil dilation [16], etc. The term gaze tracking instead refers to the estimation (and the temporal tracking) of where a person is looking at, in terms of interaction with the scene, and it is obtained by determining the 3D line of sight between the user and the target. A common practice is to consider the gaze tracker as the last block of a system that starts by localizing and tracking the eyes [17]. On the other hand, some works define gaze trackers as a subset of the eye tracker families [18].

In this arena, it is being increasingly useful to find guidance trough exhaustive survey/review articles collecting most relevant works and putting clear the pros and cons of existing techniques, also by introducing a precise taxonomy. This kind of manuscripts allows for researchers and technicians to choose the better way to move towards their application or scientific goals. Valuable attempts to give a review of the existing literature have been provided in [19,20]. These manuscripts crystallized the state of the art in 2009 and 2013, respectively, and they gave a holistic view of hardware, user-interaction, eye detection, and gaze mapping techniques. Afterward, a plethora of new works have appeared in the literature, and then it has become more useful and fruitful to focus on every single aspect of the process rather than on the entire algorithmic pipeline. This led authors in [21] to review methods for eye region and pupil detection and localization, whereas, in [22], the gaze mapping functions based on interpolation or approximation that determine, starting from the pupil glint, the coordinates on a screen, are summarized. Works dealing with full-face appearance-based gaze estimation have been discussed in [23] and an overview of key technologies of gaze tracking has been proposed in [24] instead. Insight into the issues related to algorithms, system configurations, user conditions, and performance has been finally provided in [25]. However, the recent advancements reached in computer vision, and more generally in artificial intelligence, boosted gaze tracking technology than any other scientific field. Unfortunately, how these great advancements in computer vision and pattern recognition have impacted gaze estimation and tracking has not been analyzed yet. This paper attempts to fill this gap and it also tries to give a broader analysis about gaze tracking that brings to a new taxonomy (extending the consolidated ones), established by considering gaze tracking as a more exhaustive task that has to estimate gaze targets from different points of view:

- from the eye of the beholder (first-person view);
- from an external camera framing the beholder’s;
- from a third-person view looking at the scene where the beholder is placed in; and,
- from an external view independent from the beholder.

Active researches and the most relevant advancements are introduced and discussed, while taking the aforementioned innovative points of view on this largely debated scientific topic into account. It should be clarified that, in this work, the systems that use active illumination techniques will not be reviewed, unless they provide some innovation in terms of the computer vision technique, or in the case of more complete architectures where infrared only represents a fraction of the contribution. Indeed, this is a common and well-established technology, already on the market and with many patented approaches, although they still present their own specific challenges to be addressed and some research lines are still open, especially for improving electronic lighting components [26] Systems that use active illumination techniques have not received a dramatic improvement from the computer vision perspective, indeed, in our opinion, the summarization in [20,25] can be considered to be still valid. However, it is worth noting that this dyadic view (systems using active lighting vs systems not using active lighting) is relatively recent with respect to the time in which the early gaze tracking solutions appeared. This is a consequence of the progress of computer vision and its successful exploitation in many assistive fields, as opposed to older solutions based on active lighting that anyway continue to be the reference point in terms of precision. Thus, in Section 3, both categories of solutions will be considered, highlighting the moment and how the evolution of gaze estimation based on computer vision, the main subject of this manuscript, has progressively led to the search for solutions that are based on the absence of active/near-infrared illuminators.

Summing up, the main contributions of this document are:

- an update of existing literature on methods based on computer vision, aiming at inferring the gaze;
- a critical review of the impact of deep learning on this research area; and,
- a new broader analysis of gaze tracking approaches from different perspectives.

In the rest of the manuscript, at first, Section 2 gives a quick overview of the terms used in the literature and it clarifies some possible ambiguities. Subsequently, Section 3 shortly introduces the reader to the history of eye/gaze tracking technology, whereas, Section 4 describes the methods followed to select papers/links. The subsequent Section 5 provides a scheme to classify gaze tracking techniques and it introduces an innovative taxonomy. Each branch of the proposed scheme is analyzed, and related works reviewed and discussed, in Section 6 and Section 7. Afterwards, Section 8 overviews available related datasets. Metrics and ways to evaluate the different solutions from both functional and non-functional perspectives are introduced in Section 9. Afterwards, the new challenges and a glimpse of future research directions are discussed in Section 10. Section 11 concludes the document.

## 2. The Need for Standardizing Terminology

In the literature, there are different ways to refer to the problem of estimating the human gaze from images: eye detection, eye tracking, eye localization, gaze estimation, gaze tracking, and point of regard are often used almost interchangeably and this can, sometimes, confuse the reader and hinder focusing the actual problem. It is therefore important, as a first step, to clarify this terminology issue.

Eye detection/localization refers to the problem of identifying whether human eyes are in the image or not. If eyes are detected, two bounding boxes are placed around the estimated regions containing the eyes [9]. The detection of the eyes can provide evidence of the engagement in human-computer interfaces, driver drowsiness, or can be just a preliminary step to speed up advanced processes in security and medical systems [27]. Eye detection is also required as a preliminary step in face recognition, facial expression recognition, face pose estimation methods, and face tracking [28]. Detection/localization outcomes are, in general, the pixel coordinates of the centers of the eye pupils [29].

Eye segmentation is related instead to the extraction of different parts of the eye (pupils, iris, eyelids, …). Precise segmentation of eye parts is mainly required for biometric or medical purposes. Generally speaking, the outcomes of the eye segmentation are the labels that are associated to each pixel in the eye region [30].

Finally, eye tracking aims at analyzing how the eye or its parts change their positions over time. This can help, for example, to analyze controlled and uncontrolled movements (saccadic movements) or to diagnose visual or neurological diseases [31].

Gaze estimation deals with the reconstruction of the line of sight, i.e., gaze direction in a single image. It can leverage different kinds of information (head pose, face geometry, facial expressions, scene contents, eye model and localization, human-objects relationship, camera parameters, and so on). Hand-free interaction is an important application for gaze estimation [32]. Gaze estimation can lead to many applications if the output is continued over time. In that case, the problem is referred to as gaze tracking and it can be exploited to analyze visual exploration paths and target fixations that provide useful support, for example, in assistive technologies and audience measurement [33]. Finally, the point of regard (PoR) is defined as the point at which the eye is looking. Notwithstanding the above definition, in many applications, the target is represented by an area, an object in the space (two-dimensional (2D), or three-dimensional (3D)), or the point/area in a screen. The aforementioned cases can be modeled with planes (plane-of-regard [34]), like in [35,36], or with bounding boxes that model the objects of interest [37,38,39]. Recently, 3D point clouds have been employed to estimate the gaze target [40]. Additionally, fiducial markers have been employed to map the gaze estimated from a head-mounted display to a precise area of a 3D object [41].

In the remainder of the document, we will use each specific term basing on the aforementioned definitions.

## 3. Short History of Eye Tracking Technologies

Early recognized studies about eye movements were based on naked-eye observations. In particular, it is common to fix the origin of eye tracking studies with the work of Louise Émile Javal, who analyzed, in 1879, movements of persons while reading a text, pointing out quick movements (that he defined with the word saccades) alternated with fixations. Anyway, the rapid sweeps of the eyes had already been observed by Wells in 1792 by using an afterimage technique and then illustrated by Crum Brown in 1878 [42]. In 1908, Edmund Huey [43] built a device that could track eye movement during the reading process. This is considered to be the first eye tracker and it consisted of an aluminum pointer connected to a contact lens. His studies are still considered to be a valuable contribution to the analysis of reading.

Beyond mere visual observation, all of the initial methods for tracking the location of eye fixations were quite invasive, involving direct mechanical contact with the cornea [44]. For example, electrooculography relied on electrodes mounted on the skin around the eye to measure the difference in the electrical potential originated by a movement of the eye. Other methods required the wearing of large contact lenses that covered the cornea and the sclera with a metal coil embedded around the edges of the lens; the fluctuations in an electromagnetic field caused by the metal coil, which was moving with the eyes, were used to measure the movements [45].

In the meanwhile, early non-invasive studies were proposed: already a few years before the publication of the work of Huey, Dodge, and Cline had invented the photocronograph, a device to measure the velocity of eye movements using a 5×7 bellows camera that was taking photos of the eyes. The headrest was fitted with an attachment for holding a narrow strip of white cardboard, which was illuminated by direct sunlight from behind the subject. The eye movement was monitored by measuring the light reflected from the cornea [46]. Although only horizontal speeds were recorded, it represents the first attempt of achieving a “remote sensing” device for eye/gaze tracking.

In 1905, Judd, McAllister, and Steel recorded the eye movements in both directions, with a Kinetoscope that recorded data on a film in a non-invasive way [47]. Additional advances in eye tracking systems occurred during the first half of the twentieth century when it became possible for the first time to take two photographic recordings simultaneously: this allowed for creating the first two-dimensional records of eye movements [48,49]. In 1931, the Taylor brothers (Earl, James and Carl) created the Ophthalmo Graph and Metronoscope [50,51]. The first one measured reader scans and fixations, while the latter was used to teach efficient reading techniques. With further technology advancements, eyes were grabbed singularly and the output recorded in a film reel, creating the first two-dimensional scan paths of subjects looking at images [52]. Please refer to [53] for more details.

The first modern head-mounted eye tracker is instead dated 1948 [54]: it carried a mouth plate that fits on to the teeth of the subject (to hold the apparatus), which makes it possible to store eye data, even in the presence of head movements.

A milestone work in the study of eye movements is dated 1967, when Yarbus [55] tried to determine how the human eye examines complex objects. A few years later, the scanpaths theory proposed by Noton and Stark [56] extended knowledge about scene exploration.

From the 1970s, the eye was mainly scanned with a television camera, detecting and localizing distinct features to detect eye movements. Because these methods were sensitive to light contrast, the most natural solution was to search for the limbus, i.e., the boundary between sclera and iris, also with the help of artificial lights and lenses. This leads to a new modern way to infer the gaze, focusing on the analysis of eye movements based on the reflections of the vector between the cornea (the outer-most optical element of the eye) and the pupil, namely the pupil center corneal reflections (PCCR) technique. Two examples of seminal contributions introduced in those years are the “bright-pupil” technique [57] and the dual Purkinje image [58]. In the first work, an illuminator was placed close to the optical axis of the imaging device, lighting up the pupil. Purkinje images are instead four (at least) images that are formed on the eye due to the reflection with a light source. The first image corresponds to the corneal reflection. When a user looks at one position in the three-dimensional space, the positions of the first and fourth Purkinje images change due to the lens accommodation of the human eye [59]. If the observed point is far, the lens becomes closer, but the distance between the first and fourth Purkinje images then becomes longer and the pupil size gets larger, which provides a way to infer the PoR. In the work just mentioned, the first and the fourth images were used. The technology exploiting the analysis of eye reflections gradually became the most widespread and, at the beginning of the 1980s, it was enhanced and standardized by using active light sources to enhance eye detection and tracking [60]: this additional light (often in the infrared bandwidth) was directed towards the eyes, causing detectable reflections in both the pupil and the cornea. From that moment, these reflections—the vector between the cornea and the pupil—started to be tracked by an infrared camera, with the arising of many companies (e.g., Tobii (https://www.tobii.com/), EyeSee (http://eyesee-research.com/), SR Research EyeLink (https://www.sr-research.com), Smart Eye (https://smarteye.se/)) that provided PCCR-based eye trackers that have been employed in both commercial and scientific applications.

Starting from the early 2000s, RGBD cameras simplified the setup of the eye tracking device and relaxed functional constraints, however, at the expense of accuracy [61]. Currently, because of its lower cost, smaller size, and ability to perceive depth in 3D scenes, the RGBD camera by Microsoft Kinect (https://developer.microsoft.com/en-us/windows/kinect/) has been widely applied in numerous fields including eye tracking [62]. Other off-the-shelf depth sensors that have been successfully used to infer gaze are ASUS Xtion Pro Live (https://www.asus.com/us/3D-Sensor/Xtion_PRO_LIVE/) [63] and Intel RealSense (https://www.intelrealsense.com/) [64].

Another relevant technology for gaze tracking relies on eyeglasses that also exploit active infrared lighting sources in order to estimate eye directions by exploiting the corneal reflection [65].

Sometimes thermographic cameras, i.e., devices that create an image using infrared radiation, have been used. The underlying idea for gaze tracking is that the surface temperature of the cornea is colder than the limbus, but thermal cameras are still expensive and it is an area of little pursued research [66]. In general, the above technologies could be coupled with contour equipment to increase their accuracy: examples are chin rests, to restrict head motion, or even more cumbersome equipment, like special contact lenses [67].

It is worth noting that the above technologies rely on hardware configurations, difficult to reproduce, often expensive, and patented.

However, in the last 20 years, computer vision and machine learning have made progress from a methodological point of view and, at the same time, optical sensors have become increasingly advanced (high spatial and temporal definitions) and cheap. This encouraged researchers to make the effort to automatically extract knowledge from visible light images moving, this way, systems complexity from the hardware to the software side.

Image processing began then to be applied to the eye image [53] and, since then, a plethora of new methods and systems performing gaze estimation have been introduced. The ability to extract higher-level semantic information from available raw data thanks to advanced machine learning strategies allowed for gaze tracking to start playing a key role in different applications [68,69,70,71,72].

This historical evolution might be the reason why, as stated in Section 1, all of the existing related survey/review documents rely on methodological, technological, and application points of view, but they are not specifically focused on computer vision aspects. Instead, the next section will introduce a new taxonomy of gaze tracking methods from the computer vision perspective.

**Figure 1:**
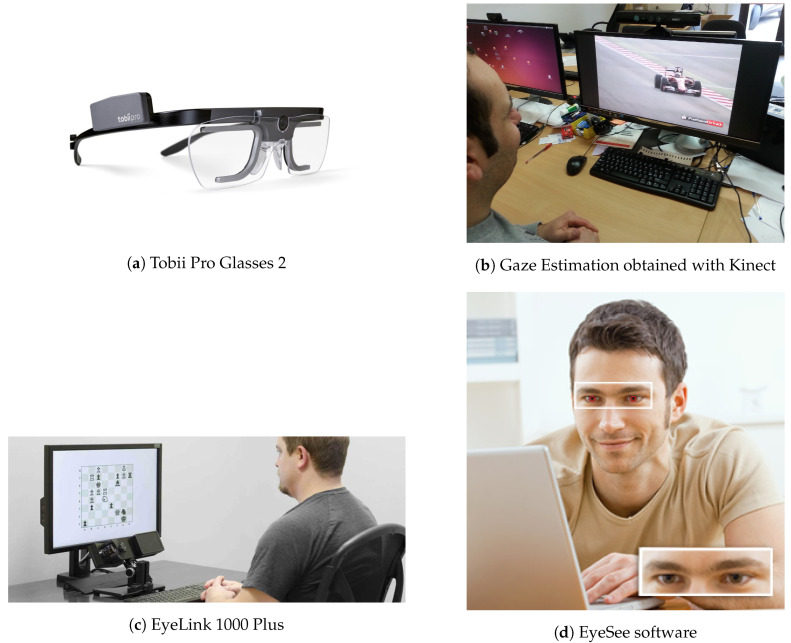
Four examples of gaze tracking systems.

Four commercial and academic solutions for eye/gaze tracking are shown in Figure 1. The device at the top left of the image (Figure 1a) is the Tobii Pro Glasses 2, a wearable eye tracker that leaves the freedom to the user to move his head in real-world settings. It consists of a full HD wide-angle camera (1920×1080 pixels at 25 fps) that grabs the scene, plus two cameras for each eye. Additionally, a gyroscope, an accelerometer, and a microphone are integrated into the sensor. Gaze can be sampled at 50 or 100 Hz, and its weight is about 350 grams. It represents a very precise solution, but also an invasive and expensive product. Figure 1b reports, instead, a less precise, but much cheaper solution, since the gaze is extracted by processing data coming from the Microsoft Kinect sensor [73]: in this paper, the authors propose a geometric method (introduced later in Section 7.1) to infer the gaze and to apply it for soft-biometrics identification purposes. The device in Figure 1c is the EyeLink 1000 Plus, a very precise eye tracker system that comprises an ad-hoc camera, a host PC, and a response device. A set of camera mounts is also available depending on the specific application (to constrain the head, or to operate with a touch screen, etc.). It typically operates at a range of 40–70 cm and at a frame rate of 2000 Hz. Lastly, Figure 1d reports a webcam-based eye tracking solution. The company targets applications related to market analysis insights and digital signage, also extracting facial expression information to track user preferences in a more complete way.

## 4. Method

As stated in Section 1, this research started from the consideration that no survey document describing the impact of recent great advancements in computer vision on gaze tracking was provided. The goal was then to collect works leveraging recent computer vision methods to improve gaze tracking in terms of accuracy, computational load, reproducibility, human intervention level, and so on. The following of this section briefly describes the methodology that was applied to selected papers, links, and datasets for the preparation of this survey. The method did not exclusively rely on keywords search in order to provide the largest coverage of the subject and considering the ambiguity behind the different terms used in the literature (Section 2). This would surely have been the simplest method, but it would have led to possible methodological discrepancies, with the consequence of unforgivable omissions. This survey started, instead, by a full methodological exploration of the most outstanding scientific content databases of peer-reviewed literature (Scopus (https://www.scopus.com/home.uri), PubMed (https://pubmed.ncbi.nlm.nih.gov/), Web of Science (https://clarivate.com/webofsciencegroup/solutions/web-of-science/), etc.), and from the technological scouting reported in Section 3. The exploration relied on the terms in Section 2, but they merely represented the entry points to discover the huge extracted literature. The authors’ background drove the aforementioned tasks to build the innovative taxonomy that will be introduced in Section 5. Once the taxonomy has been fixed, for each leaf of the tree, a deep analysis of the relevant works has been carried out when considering, as selection criteria, the following:

1. the introduced advancements in terms of knowledge, i.e., how much the proposed approach contributed to the definition of the taxonomy branch;
2. the impact of the method into the literature, i.e., how many works make use of it;
3. the publication date, giving the priority to works published in the last decade; and,
4. only for recent works, since the first two criteria cannot be directly applied, the potential breakthrough from the application and technological points of view and the rank of the computer science conference and journal.

## 5. Taxonomy of Computer Vision-Based Gaze Tracking

**Figure 2:**
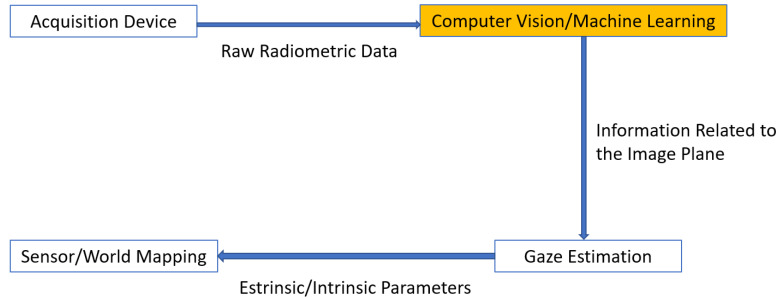
A block diagram of a typical gaze estimation framework.

Any system performing gaze tracking relies on a technological and methodological framework that can be schematized, as in Figure 2.

In light of the above scheme and considering what it has previously been reported, a straightforward classification of eye/gaze tracking approaches could rely on the underlying exploited hardware technology, i.e., the family of employed acquisition devices. This might be very easy to do since it depends on discernible features about which, besides, it is quite easy to collect technological information. An unexplored and maybe more effective way to categorize gaze tracking devices focuses on computer vision and machine learning modules involved to step up from raw radiometric data to semantically more significant information about the line of sight.This kind of categorization will drive the investigation in this paper.

The following step that maps the line of sight into a 3D gaze vector has been taken into consideration too, but only if strictly related to the methodology itself, i.e., if the mapping is built depending on the development of the computer vision algorithmic pipeline. The relationship between world coordinates and image plane coordinates has not been examined, instead, if it just applies well-known mapping techniques coming from the literature [25].

**Figure 3:**
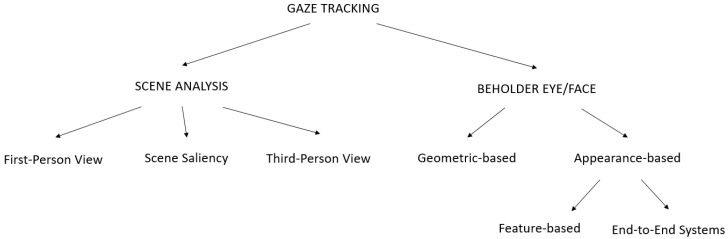
The new proposed taxonomy for categorizing gaze tracking techniques. It considers two different branches related to what is framed in the acquired image: the eye/face of the beholder or the scene observed by him.

From this perspective, gaze tracking approaches might be categorized depending on what is framed by the acquisition device. The first category consists of approaches that estimate gaze by looking at the surrounding scene (Scene centered). Approaches that process images framing the subject’s face or part of it belong to the second category (Beholder centered). In turn, three subcategories fall in the case of scene centered analysis: the approaches that exploit the first-person view, i.e., through a direct estimation (e.g., by framing the scene from wearable devices such as glasses or helmets); approaches that estimate gaze using an environment-mounted camera, i.e., a *third-person viewpoint*; approaches that estimate gaze from the features in the scene (indirect estimation), i.e., saliency. In the beholder centered category there are two different ways to proceed: some works rely on a model of the eye (geometric-based) and some others that focus on face/eye appearance (appearance-based). Appearance-based methods can resort to feature-based or not (end-to-end estimation). The proposed classification is summed up in Figure 3. In the following, each leaf of the tree represented in the figure will be analyzed in detail.

## 6. Gaze Tracking by Scene Analysis

This section collects works that attempt to estimate gaze by directly observing the potential targets in the scene. As already introduced in Section 5, three different subcategories can be identified when considering if the methods rely on a first-person view (e.g., by framing the scene from wearable devices such as glasses or helmets), on features of the scene (by only considering scene dynamics and/or objects’ saliency) independently from the point of view, and on a third-person view (using an environment-mounted camera in which the beholder is also framed in).

### 6.1. First-Person View

This section reports those works leveraging implicit cues that exist in camera wearer’s behaviors. In this egocentric setting, which imitates how humans perceive and analyze scenes, gaze prediction can be used to achieve user-centered goals, such as segment task-relevant foreground objects, recognize actions and gestures, understand the surrounding scene, monitor human-object interactions, understand and enhance the quality of living, create daily life activity summaries, and infer the camera wearer’s identity or body pose. This is a relatively new field of research that has emerged with the increase of the use of wearable and immersive computing devices, enabling the acquisition of images and video from a first-person perspective.

The seminal work dates back in 2013 [74]. The proposed approach is specifically designed for predicting gaze in egocentric vision during object manipulation tasks. For this reason, it relies on implicit cues that are provided by first-person, such as hand location and pose, or head/hand motion. The key idea is that there exists strong coordination of eye, head, and hand movements in the object manipulation tasks, and, thus, these coordinations can be exploited for predicting gaze in the egocentric setting. To do that, it can be built a graphical model that accounts for eye-hand and eye-head coordination and combines the temporal dynamics of gazes. The method benefits from using the strong egocentric cues (head, hand, and eye coordination) for gaze prediction and then it can be only exploited if these data can be extracted (hands have to be visible) and accurate (the optical flow that authors use for motion estimation may fail in case of unreliable matching between points in different frames).

Convolutional neural networks (CNNs) broke in by the work in [75], in which a recurrent convolutional neural network (RCNN) and a fixation state predictor generate an attention map for each frame taking into account previously fixated regions and head motion. Two assumptions drove the development of this approach: firstly, a person’s gaze tends to be located on the same object during each fixation, and a large gaze shift almost always occurs along with large head motion. Secondly, patterns in the temporal shift between regions of attention are dependent on the performed task and they can be learned from data. The method is motivated by the fact that the behaviors of the first-person provide strong cues for predicting the gaze direction. This can directly lead to the conclusion that this approach is not suitable when abnormal behaviors have to be detected or analyzed, e.g., for assistive or diagnosis purposes, whereas it perfectly suits diarizing of daily activities.

The most common approaches predict gaze on the basis of the framed objects (e.g., Object Gaze algorithm in [76]). Once again, deep learning is largely exploited to recognize objects [77] and, to this aim, deformable convolutions [78] are very well suited, since they can model large unknown transformations that happen in egocentric vision. The feature maps from a previously trained object detection model along with the object detection results are aggregated to make a prediction regarding the gaze point. Classification and regression strategies are finally exploited to move from an initial coarse prediction to a finer one. If egocentric gaze analysis is finalized to check fixations on a few predefined static objects, then the detection can leverage region-based detectors (instance segmentation) to achieve real-time and accurate outcomes [79]. For example, the study in [80] develops and tests an automated system that maps the gaze fixations of workers on a construction site and analyzes it to determine their visual attention distribution. The proposed method uses RCNN and transfer learning [81] to detect predefined objects (i.e., hazards). Workers wear glasses that capture videos of the worker’s view while they move in a road construction site. The study paves the way for automating personalized safety training by combining computer vision techniques and eye tracking technology. Object-based approaches are generally limited in the presence of several different objects in the scene and when the head moves very rapidly, since objects appear blurred and their detection is more challenging. The incorporation of temporal data or the use of cameras that are capable of recording videos at a higher frame rate might fix this issue, but could adversely affect the speed of the model.

An unsupervised computational model that draws inspiration from cognitive psychology models of human attention and event perception has been introduced in [82]. The basic building blocks of the representation are atomic components obtained by dividing the input image into an M×N grid. Atomic components are features extracted from egocentric videos and are used to estimate the gaze at each time step through a pattern theory representation that, among all, defines an energy function: gaze position corresponds to the grid cell with maximum energy. These features can be appearance-bases, such as RGB values, motion features, such as optical flow, or deep learning features. Temporal consistency is enforced through an ordered weighted averaging aggregation operation of configurations across time. This unsupervised method appears very promising, but it is still in its infancy, and additional investigations are required in order to understand the actual capability to enable gaze prediction without training.

Finally, a strictly related problem extends the conventional gaze prediction problem to future frames by no longer confining it on the current frame (this task is referred to as gaze anticipation in the literature). To solve this problem, generative adversarial neural network based models are exploited [83]. From the methodological point of view, the most interesting contribution concerns a two-stream 3D-CNN developed to explicitly untangle foreground and background motions in egocentric videos. Motion cues are then learned and used to model gaze prediction in an end-to-end way. From the application point of view, gaze anticipation could be very useful in dyadic conversations or HRI scenarios to predict whether gaze will be averted in the near future.

### 6.2. Scene Saliency

The objective of visual saliency prediction is to estimate locations in an image that attract the attention of humans looking at it.

Starting from the seminal work of Itti et al. [84], visual saliency prediction has been extensively studied in the literature. In particular, in the last decade, saliency maps have been largely exploited to learn the relationships between the gaze probability maps and eye images in order to obtain gaze estimators that exempt users from active calibration as in [85].

The great improvements in saliency maps estimation drove to the analysis of image/video features to directly predict eye fixation and gaze direction instead of using them as a supporting tool during the calibration phase. Generally, most of the existing saliency prediction models are based on bottom-up, top-down, or hybrid approaches. Bottom-up approaches employ low-level image features, such as intensity, color, and orientation to predict visual attention, while top-down approaches take into consideration high-level features of the scene (such as object/human relationships) and the context. Hybrid approaches combine low-level and high-level features to improve performance, allowing for recent applications to predict video attention during natural interactions of the user with a smartphone [86]. With advances in deep learning, new saliency-based gaze tracking models are continuously proposed, but they are most exploited to learn salient regions in order to predict eye fixations and then eliminate explicit personal calibration while using PCCR based eye trackers [87]. In the following, some works specifically devoted to the gaze tracking via saliency map estimation are reported. A biologically inspired bottom-up model can be found in [88]. The input of the model is a video that is split into spatial and motion information. This split comes from biological reasons, since the retina and the cortical cells are sensitive to the different spatial frequencies for the different pathways. Some basic features are considered: signal orientations and spatial frequencies for the static saliency, and the module of motion for the dynamic saliency. At the output of each pathway, a saliency map is extracted. The static and dynamic saliency maps are fused to create the spatio-temporal saliency map that predicts eye movement during free-viewing of videos. The model works well under certain conditions, but it does not consider how spatially varying sampling of the retina, depending on eye positions, can affect the gaze prediction. Moreover, color changes are not included into the model parameters.

Authors in [89] predict focus of attention when viewing, on a 2D screen, videos that contain a depth map (RGBD data). The method employs a generative convolutional neural network to reconstruct saliency for each frame by implicitly learning the gaze transition from the previous frame. The network was trained to predict the saliency of the next frame by learning from depth, color, motion, and saliency of the current frame. Depth-aware saliency maps are very accurate on natural scenes, but their effectiveness on close-up acquisitions might be limited. Additionally, the inability to handle separately RGB and depth data (not always available) represents another limit.

To the aim of accurately mimicking human attention, some authors introduced more complex deep neural models that are also able to take into account additional factors. For example, how the emotion-evoking objects affect the attention, has been evaluated in [90]. The basic idea is that emotion-evoking objects receive attention favor, especially when they co-occur with emotionally-neutral objects, and this favor varies, depending on the different image complexity. Based on this empirical hypothesis, the authors have designed a deep neural network for human attention prediction, which allows the attention bias on emotion-evoking objects to be encoded in its feature space. This “automatic” capture of the attention is supported by research in neuroscience, but the improved metric to assess human attention does not comprise all of the emotion-evoking objects, so it has to be carefully handled in the case of specific applications (especially in clinical settings), since this discrepancy can cause drifts on saliency outcomes.

A very outstanding approach for fixation prediction has been introduced in [91], in which a Convolutional Long Short-Term Memory (CNN-LSTM) that focuses on the most salient regions of the input image to iteratively refine the predicted saliency map is used. The model is able to predict high saliency values on people, faces, objects, and other predominant cues. It also produces good saliency maps when images do not contain strong saliency regions, such as when saliency is concentrated in the center of the scene or when images portray a landscape. Of course, it needs a temporal evolution to refine saliency, thus it is not suited in application contexts in which gaze has to be predicted on static scene.

Finally, there is a line of research exploiting saliency to extract personalized parameters of the gaze estimation function without using explicit training data. In [92], this is achieved by combining saliency maps with the synchronized eye images. Taking a synchronized set of eye images and video frames, the method trains the gaze estimator by regarding the saliency maps as the probabilistic distributions of the gaze points. Unfortunately, the head movements affect the gaze estimation, which makes the approach not mature for real world applications. In [93], it is introduced a framework that utilizes saliency information in the visual content to transparently adapt the gaze estimation algorithm to the user, without explicit calibration. An algorithm to transform a saliency map into a differentiable loss map that can be used for the optimization of CNN-based models is introduced. In practice, an extra fully-connected (FC) layer is added at the end of a CNN that predicts the user’s gaze position and this can be exploited by any CNN-based gaze estimator. The additional layer is then fine-tuned and the mean squared loss is replaced with a custom map loss built by using videos annotated with ground truth saliency data. This is a very truly enlightening idea that can also handle imprecise annotations in ground truth data.

### 6.3. Third-Person View

**Figure 4:**
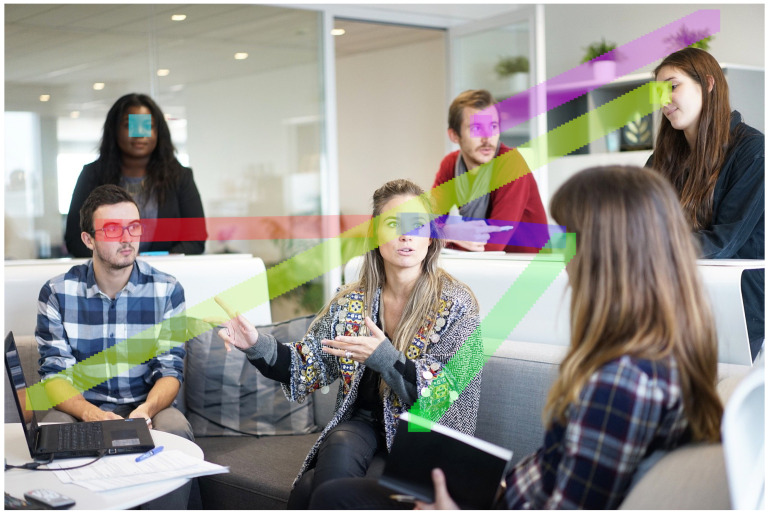
An example of a third-person view in which several people are in the scene. Differently from the gaze estimation from a single user facing a sensor, here the sensors are ecologically placed in the environment. The task comes with many challenges given by the necessity to consider postural, relational, semantic, and contextual information.

This section collects the works estimating gaze using an environment camera, i.e., from a third-person viewpoint. Sometimes, in the literature, this topic is referred also as attention modeling, object/person referring or gaze following, depending on the application context in which it is put in place. In Figure 4, an example of a third-person view in which several people are in the scene is illustrated: understanding where each person is looking at is the aim of this research area. The outcomes of this task can provide an online and implicit way of investigating the dynamics of human interactions, and between human and machine. Moreover, the relevant features that capture the observer’s attention according to the amount of information the observer has available can be pointed out.

An initial contribution in this research field has been given in [94]. The aim is to track the attention of the communicating party from a single low-resolution camera. This is a crucial task in many application fields and it plays a central role in establishing joint attention in HRI contexts. The proposed approach learns gaze direction from head pose estimates and it then exploits saliency to fix on interesting objects at first, and then to reduce the uncertainty in the estimation of the gaze direction. The approach works without taking into account body postures, the eye appearance, and the scene depth, so it can suffer in real application contexts, e.g., with freely moving humans in heavily cluttered scenes, i.e., with several salient objects that are present at the same time.

A work that paved the way towards advanced approaches was, instead, the one in [95], where a deep neural network was trained to detect, in a static image, the location of the head, to follow the gaze of the person, and to identify the object being looked at (such as televisions, balls, or food). The architecture of the proposed deep network for gaze-following has two main components: the saliency pathway to estimate saliency and the gaze pathway to estimate gaze direction. Images with several salient objects are naturally handled, since the problem has been posed as a classification task that provides several outputs (related to grid cells) with associated confidence values.

Another important contribution has been provided by the work in [96]. The proposed model leverages a multi-task learning framework and it takes three inputs: the entire image, the subject’s cropped face, and the location of the face of the subject whose attention must be estimated. Given this inputs, the model estimates the subject’s gaze angle in terms of yaw and pitch degrees (the “where” component of the visual attention), the subject-dependent saliency in terms of a heat map (the “what” component of the visual attention), and how likely the subject is fixating at the estimated gaze target in the scene (the overall “strength” of the visual attention). The scene and the face images go through separate convolutional layers and, in the very last layer, the final feature vectors for these two tasks are combined to estimate how likely the person is actually fixating at a gaze target within the observable scene. A cross-domain learning approach where the model learns partial information relevant to each task from different datasets has been exploited. This is the first attempt to solve the gaze estimation problem from a third-person view, even in scenes containing several persons interacting with each other. The limitation is given by the huge amount of knowledge to be extracted from data and then annotated datasets tailored for this problem are still a requirement.

A different way to get around unsolved problems is to exploit temporal cues (i.e., using videos instead of a static image) and/or additional prior information. This is what happens in those works investigating the problem of object referring, i.e., to localize a target object in a visual scene coming with a language description that is the prior information. The network model in [97] uses text language, gaze estimates, visual appearance, motion features, and depth features to localize the object being referred. The model is based on the Spatial Context Recurrent ConvNet model and it unifies three LSTMs in order to integrate information from language expressions, global visual context, and local object content.

The problem of attention targets in videos has also been addressed in [98], where authors also try to correctly handle the case of out-of-frame gaze targets. This is achieved by introducing a novel architecture that models the dynamic interaction between the scene and head features and infers time-varying attention targets. Spatial and temporal reasoning is used to improve the accuracy of target localization. This is achieved through a convolutional layer that focuses on the scene region towards which the head is oriented. The temporal dimension is addressed through the addition of a CNN-LSTM. The approach is very promising and it could be effectively exploited, for example, by extracting clinically-relevant gaze behavior without wearable cameras or eye trackers.

Under the taxonomic term of third-person view fall also some latest works that deal with joint attention. Joint attention is the shared gaze behaviour of two or more individuals on an object or an area of interest, and it has a wide range of applications, such as human-computer interaction, educational assessment, treatment of patients with attention disorders, and many more. A methods that learn joint attention in an end-to-end fashion has been introduced in [99]. It uses saliency augmented attention maps and two novel convolutional attention mechanisms that select relevant features and improve joint attention localization. The network only takes raw images and detects faces and their respective co-attention locations. It works without using any temporal information and other dependencies, such as a face detector or head pose/gaze estimator.

Joint attention is also the central topic in [100], where a Bayesian generative statistical model for temporal scene understanding using probabilistic graphical modeling notation is introduced. The model captures the joint probability of camera parameters, locations of people, their gaze, what they are looking at, and locations of visual attention. In this problem, the number of people in the scene and the number of extra objects that draw the attention are both unknown and need to be inferred.

To infer shared attention in social scene videos, a spatial-temporal network that explicitly leverages human gaze direction, target region candidates, and temporal inter frame constraints has been introduced in [101].

The identification of joint attention mechanisms in a triadic interaction (child-adult-humanoid robot) has been proposed in [102]. Here, a depth camera is non-intrusively mounted on the top of a room to detect the arising event performed by a humanoid robot and, subsequently, the eventual joint attention mechanism, analyzing the orientation of the head of the persons in the scene.

Finally, the problem of following gaze across views has been analyzed in [103]. Given one view with a person in it and a second view of the scene, the model has to estimate a density for gaze locations in the second view. This is a more challenging problem that requires simultaneously estimating saliency, gaze and geometric relationships between views.

## 7. To the Beholder’s Eye/face

**Figure 5:**
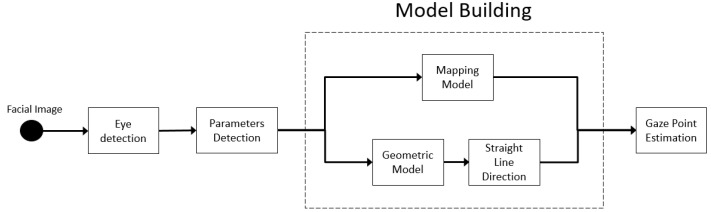
A block diagram of a typical gaze tracking solution from the analysis of the eye/face.

In this section, gaze tracking systems working by processing data coming from a sensor pointing towards the user are analyzed. Although each work has its proper peculiarities and uses a specific model, it is possible to derive a generic schema that is introduced in Figure 5. In this representation, it is possible to derive how, first of all, the eyes must be detected and tracked. Eye and pupil detection are full-fledged independent and well-investigated problems in the literature [7,104], whose definitions have been given in Section 2. Herein, some references and useful information will be provided to better guide the reader to the analysis of each component of the proposed schematization. Generally speaking, it is possible to divide eye tracking solutions into intrusive and non-intrusive [20,105]. Examples of intrusive solutions are electrooculogram [106] or scleral coil [107]; quite precise and less intrusive systems can employ active infrared (IR) illuminators and the PCCR technique [108,109]. In the last two decades, impressive results in terms of precision have been obtained using computer vision techniques: they process images acquired by consumer cameras achieving completely non-invasive estimations [110,111,112,113,114,115,116,117,118,119,120].

Data acquired from the eye detection step are often used together with other information extracted from the face to estimate a set of parameters that will be used by a model in order to extrapolate the PoR on the scene or a 3D line of sight. Here, it is possible to distinguish between geometric-based gaze estimation, which relies on a model of the eye, and appearance-based methods, which focuses on face/eye appearance.

In the case of geometric-based methods, the model consists of a 3D straight line between the observer’s eyes and the PoR, expressed in physical units; as a consequence, the set of parameters aims at estimating such 3D straight line. Examples are the head pose in terms of yaw, pitch, and roll angles [121], the distance between the user and the camera, the 3D position of the pupil center and/or eye corners, and the eyeball center.

In the case of appearance-based methods, the model computes a direct mapping between the eye/face patches and the PoR, acting as a regressor (or as a classifier). The parameters here usually consist of the output of a feature extractor step computed upstream on the eye/face patch.

The linearity of the aforementioned scheme can offer interesting variations. In some case [122], geometric parameters can be used to compose the feature vector that is then used to obtain a direct mapping with the PoR: in our opinion, this potentially hybrid approach belongs to the category of appearance-based methods, because the output of parameter extraction represents full-fledged handcrafted features that are extracted to map a face/eye image with a gaze point, employing machine learning downstream. It is also important to note that the way the presence of the eyes is detected (eye detection/localization step) can differ from the gaze estimation step approach from a taxonomic viewpoint, but the gaze estimation method should still be classified, depending on how the PoR is effectively estimated, and not how the eye and pupil are extracted.

**Figure 6:**
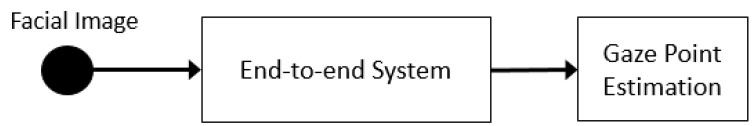
Scheme of end-to-end system from the analysis of the eye/face.

Lastly, in particular with recent advances in deep learning [123], many works have proposed an end-to-end map between the face/eye regions and the PoR [23]. Because such systems present a direct mapping between an image and a position, in this work, we classify them as appearance-based methods. In this case, the parameter estimation step is omitted and the block diagram simply becomes as represented in Figure 6. It is worth observing that the eye detection step is missing in the scheme, given that the image of the whole face [124,125] or just the eventual rough estimation of the eye regions [126,127] are provided as input.

In the following, the main computer vision techniques used in the state of the art to infer the gaze will be detailed and discussed for each of the categories under consideration.

### 7.1. Geometric-Based Gaze Estimation

Geometric-based methods infer the gaze using a 3D model of the head and/or the eye by geometric reasoning. In particular, they detect eye shapes, like the pupil center, iris edges, facial feature pose to locate head and eye position, and their orientation in the space. This information, together with available or estimated 3D knowledge, is used to estimate the PoR by intersecting a 3D straight line with the reconstructed or calibrated scene.

A way to navigate the wide existing works is the presence or absence of a calibration phase. In fact, some works rely on a person dependent parameter estimation, thus a one-time personal calibration is necessary to obtain all of the parameters [128], while, in other cases, anatomic data [129] is either averaged [122], or person-independent constraints are introduced [130] in order to substitute the person-dependent calibration.

Apart from the presence/absence of extra person-dependent parameters, two different sub-problems must be addressed to infer gaze using a geometric method: the creation of a 3D model of the single eye and the integration of the eye(s) with the head.

In the simplest case, the eyeball is modeled as a sphere [61,73]. More complex eye models do not use a single sphere, but a fused two-piece unity [128], basing on a model closer to the medical anatomy of the eye [50]. Generally speaking, works that proposed more complex eyeball models are those that assume a precise localization of the parameters in the image, even at the price of working only on ideal conditions and/or using manually labeled data in the experimental phase. With the spread of low-cost depth sensors, automatic pipelines that introduced fewer constraints have been progressively proposed. In this case, the accuracy in terms of depth information, as well as the RGB resolution, are inadequate for estimating the parameters that are assumed from elaborate anatomic models. On the other hand, such innovation has lead to less precise, but real-time, uncalibrated, and/or person-independent gaze estimation solutions.

Once parameters that are related to the eye model are estimated in the 3D environment, the information must be integrated with the head pose in order to trace the 3D straight line. Some solutions estimate the angle between the optical axis and the 3D pupil position, and a straight line from the corneal center to the pupil position is traced [128,131]. In other cases, this model is simplified by estimating the eyeball center using the head pose vector direction [73]. The latter can provide a simplified general working scheme and will be detailed as first. In this case, if we denote with:

- $\mathrm{EyeCenter}=(x_{\mathrm{EyeCenter}},y_{\mathrm{EyeCenter}},z_{\mathrm{EyeCenter}})$: the 3D coordinates of the center of the eye, on the eye sphere surface;
- $\mathrm{EyePupilCenter}=(x_{\mathrm{EyePupilCenter}},y_{\mathrm{EyePupilCenter}},z_{\mathrm{EyePupilCenter}})$: the 3D coordinates of the center of the eye’s pupil;
- $\mathrm{EyeballCenter}=(x_{\mathrm{EyeballCenter}},y_{\mathrm{EyeballCenter}},z_{\mathrm{EyeballCenter}})$: the 3D coordinates of the center of the sphere that models the eye; it is a variable that is not visible;
- ${\mathrm{Radius}}_{\mathrm{EB}}$: eyeball radius; and,

then it is possible to integrate the head pose information, like in [73], as:

(1) $$\mathrm{EyeballCenter}=\left\{\begin{array}{c} x=x_{\mathrm{EyeCenter}}\pm \left|{\mathrm{Radius}}_{\mathrm{EB}}\cos\omega_{x}\sin\omega_{y}\right|\\ y=y_{\mathrm{EyeCenter}}\pm \left|{\mathrm{Radius}}_{\mathrm{EB}}\cos\omega_{y}\sin\omega_{x}\right|\\ z={\mathrm{Radius}}_{\mathrm{EB}}\cos\omega_{x}\cos\omega_{y}+z_{\mathrm{EyeCenter}} \end{array}\right.$$

where $\omega_{x}$ and $\omega_{y}$ are the pitch and yaw head pose angles, respectively. This way, gaze can be computed in a straightforward manner, moving the computational effort to the head pose and pupil detection steps; the solution is also independent of the specific algorithms employed to detect the 3D head pose and the positions of the pupils, representing a modular solution with possible real-time performance. From the other side, the fact that the eyeball center is not observable and can only be roughly approximated, a small imprecision can lead to inaccurate results: this often requires a post-filtering step with the effect of temporally smoothing the predictions, but this solution is only possible if video sequences are processed.

Instead, a model for estimating the optical axis for a two-sphere eyeball model is provided in [128]. Here, the approach is similar, but the straight line is traced by inserting optional parameters that are:

- $C_{0}$: corneal center;
- $K_{0}$: distance between corneal center ($C_{0}$) and EyePupilCenter; and,
- *K*: Euclidean distance between the EyePupilCenter and the eyeball center (in this case, of the biggest sphere, and denoted now with C).

Thus, the corneal center is estimated as:

(2) $$C_{0}=C+\frac{K_{0}}{K}\left(EyePupilCenter-C\right)$$

and the straight line can be traced. In such a model, a person-dependent calibration can be provided in order to correct this angle with the subjective way to see at the objects of a specific person. A way to incorporate this subjectivity in the model is to provide two additional (horizontal and vertical) person-dependent angles for the visual axis deviation in the form of $\kappa =(\phi_{\kappa },\theta_{\kappa }$) [132]. This model is simple to set up and it can also work in outdoor scenarios. One drawback of this work and, more in general of geometric models, is that it is difficult to precisely estimate the 3D parameters, and an error of few millimeters or pixels can propagate in the space, leading to highly wrong estimations. At this purpose, 3D vision, e.g., depth sensor, stereo pairs, etc., can easily provide a mapping between pixels and 3D world coordinates, with the effect of refining the precision of such methods. In [133,134], Microsoft Kinect is used to integrate 3D information of the sensor to the processing on the RGB image, while an example of a stereo pair is in [135].

The work in [136] employs RGBD information to infer gaze by a geometric model that takes both eyes into account by averaging the two single eye estimations. If $P_{g}^{L}$ and $P_{g}^{R}$ are the left and right point of gaze (intersecting the 3D straight line of the gaze with the screen, whose position w.r.t. the sensor is pre-calibrated [36]), the final point $P_{g}$ is computed as:

(3) $$P_{g}=\frac{1}{2}\left(P_{g}^{L}+P_{g}^{R}\right)$$

Authors also prove that the two-eye model averages the gazes of both eyes for a final gaze estimation that can outperform single eye methods. However, in the case of large head movements, the error in the estimation of the parameters that are related to one of the two eyes becomes larger, which affects the final estimation accuracy.

Other works focused on removing typical constraints, like the user appearance or a pre-learned user model, computational load, and the problem of calibration (user or system dependent). In this regard, examples of real-time and uncalibrated gaze estimation method that can apply to daily-life situations have been proposed in [73,137,138].

### 7.2. Appearance-Based Gaze Estimation

With appearance-based models, a set of features is extracted from patches that are related to the eye or face and it is employed to fit a model that will extrapolate the PoR. To create this mapping, cropped eye images of one or more subjects looking at known locations are used. It depends on the proposed work in the literature if one or two eyes are considered, as well as if the head can be freely moved in the scene or not.

In fact, early works assumed a fixed head pose or only allowed slightly head movements [139,140,141], while, afterward, head pose information has been integrated to allow the user to freely move the head in the scene [142,143].

Principally, achieving appearance-based gaze estimation consists of finding a mapping using two sets $T_{S_{x}}$, $T_{S_{y}}$ (for the horizontal—$S_{x}$—and vertical $S_{y}$ points of regards, respectively) of cardinality *N*:

(4) $$\begin{array}{c} T_{S_{x}}=\left\{\left(e_{i1},e_{i2},S_{ix}\right),i=1,\ldots ,N\right\}\\ T_{S_{y}}=\left\{\left(e_{i1},e_{i2},S_{iy}\right),i=1,\ldots ,N\right\} \end{array}$$

$e_{i1}$ and $e_{i2}$ are the features vectors extracted by the two eyes in the frame *i* and lie in ${I\;R}^{m}$. Given a set of correspondences between eye patch or eye features and observed well-known points (a calibration procedure), the regression polynomial coefficients array can be estimated by minimizing a cost function, minimizing the error in the training data.

#### 7.2.1. Feature-Based Methods

Apart from linear regression and its slight variations [139], a variegate set of machine learning techniques with different feature extractors have been employed in the literature to estimate the gaze. An example is given with multi-level Histogram of Oriented Gradients (mHoG) to train a Support Vector Regression (SVR) and a Relevance Vector Regression (RVR) model in the case of head-mounted camera [144], showing how low-level gradient features are well-suited to capture variations in the appearance of the eye. Authors demonstrate that, with the data used in the experimental phase, RVR works similarly, but using significantly fewer basis functions, providing a more efficient solution with higher generalization capacity if the number of calibration samples is low.

Instead, the work in [145] creates a 36-length feature vector with eye contours, iris size, and location, and pupil positions estimated using an Active Appearance Model (AAM), which represents the input for a Support Vector Machine (SVM) to classify the gaze among five different directions. Different shorter feature vectors have been used and compared, but the SVM scale tends to compress the eye region to a small interval, with the consequence of getting incorrect classifications in the case of shorter lengths. An advantage of the proposed solution is that the obtained accuracy does not depend on the camera resolution. The eye corner-iris center vector (EC-IC) is introduced as a feature vector to find parameters of polynomial regression with radial basis functions in low-resolution images in [146]. The iris is modeled as a circle, and it is found while using a two-stage solution: the first one aims at obtaining a coarse location of the iris center. Because the image gradient of iris boundary pixels will be pointing outwards, the detection of the eyes is based on the directions and the intensity information of the gradient. The peak location that corresponds to the center of the circle is found using Hough Transform Filter kernels [147]. In the second stage, the IC location is further refined using boundary tracing and ellipse fitting. Eye corners are found using Gabor filters [148]. The EC-IC vector is calculated separately for the left and right eye as the difference (in pixel coordinates) between eye center and iris center coordinates, and it is tracked over time. The regression function obtained from a calibration procedure is used to map the EC-IC vectors to the PoR in terms of screen coordinates after averaging the positions that are returned by the left and right eye models.

In [149], manifold learning is used to the feature vector made by mHoG and Local Binary Patterns (LBP). The method creates a manifold embedding for each person in the training data to learn a generic linear transformation. Dimensionality reduction for each person in the dataset is performed, and the known gaze directions and person identities are used to align the resulting manifolds across persons. The approach is linear and computationally efficient, since it results in an additional rotation of the Principal Component Analysis (PCA) results. The work shows that manifold alignment can improve the estimation performance, in particular for the case of a low number of target dimensions, showing the best performance in combination with multi-level Histograms of Oriented Gradients mHOG features and nearest neighbors for regression.

The problem of estimating the gaze of users watching at a tablet has been considered in [150]. First of all, the eyes in the image are detected by a cascade eye detector and eye regions are cropped. mHoG features are extracted and linear discriminant analysis is applied to perform dimensionality reduction on the feature vector. The output is fed to a RF regressor. Moreover, this work introduces the TabletGaze dataset (see Section 8) for the performance evaluation, and a comparison of different combinations of feature extractor and four regressors, namely k-Nearest Neighbors (k-NN), Random Forest (RF), Gaussian Process Regression (GPR), and Support Vector Regression (SVR).

Additionally, for the case of feature-based methods depth sensors have been employed. For instance, Microsoft Kinect has been employed in [151]. The main idea is to perform 3D rectification of the eye images into a canonical head pose viewpoint and scale, regardless of the actual head pose. RGBD data are exploited to track the head pose and to do a warping of the eye texture as a function of the estimated head-pose, thus obtaining head pose invariance, but at the cost of a learned person-specific 3D mesh model.

An analysis-by-synthesis approach has been proposed to infer gaze in [152]. The idea is, given an input image, to fit a 3D morphable model to reproduce a synthesized image that matches as much as possible the input. The gaze is estimated from the fitted eyeball pose parameters. The search for optimal parameters is performed defining an objective function of two weighted components: one term minimizes the difference in image appearance and the other one is a regularization term against reliable facial feature points.

More recently, feature-based works exploited deep learning capabilities to compute the mapping without the need for an explicit model, but still after the handcrafted creation of an input feature vector. For example, in [153], CNN is used to extract features and Random Forests (RF) regression is used to take advantage of data distribution in the sparse feature space.

#### 7.2.2. End-To-End Systems

It is a matter of fact that the very recent spread of deep learning [154] can be considered as the breakthrough technology for the whole field of artificial intelligence; the gaze is not an exception, where deep learning opened up to the possibility to apply end-to-end approaches. Notwithstanding, the literature presents noticeable results for obtaining an end-to-end mapping without necessarily using deep networks. For example, in [155], SVR has been directly applied to the stacked eye image pixels. A small motor-driven mirror is installed to infer the gaze without requiring active lighting and with little intrusiveness. The system is conceived for visual behavior monitoring in young children, thus the calibration is performed online in order to allow the use of non-cooperative users. Finally, and eye blinking detection module is added to avoid tracking errors. In the work in [156], multiple miniature low-resolution cameras positioned around a head-mounted setup are used. The gaze-mapping function is learned from using a small artificial neural network trained on a large dataset of eye images. These small patches (from four cameras of only 5×5 pixels resolution in the experimental evaluation) are directly fed to a small neural network composed by two FC layers with 512 hidden units and ReLU activation. The single outputs are merged in one last FC layer with 512 hidden units, and the final network prediction is provided by a linear regressor.

In the work of [126], an RF is used as a regressor to estimate the gaze in eye patches normalized, depending on the 3D head pose estimated by six facial landmarks, introducing synthetic data generation for increasing the performance. The gaze estimator is trained with an extension of random forests, learning a set of regression trees with redundant subsets of the training data. This redundancy is introduced in order to model the nature of the mixed-modal input and leads to an improvement on the estimation accuracy. Authors also introduced the UT Multiview dataset.

A seminal work that proposed a deep learning-based end-to-end system is in [157]. The eye patch is normalized like in the aforementioned work [126], but chaining the eye patch together with the head pose angles and creating an input for a LeNet neural network. In the same paper, authors propose the MPIIGaze dataset for learning the mapping with gaze coordinates. Finally, the authors compared this method with classic machine learning approaches: in both cross- and within-dataset evaluation, the CNN gave noticeable results although authors stress the condition where [126] can still outperform, making the first hypothesis on the learning capabilities of CNNs in the case of gaze estimation. From this moment, many works exploited new advancements of the deep learning era to improve gaze estimation performance.

In [158] iTracker, a CNN that can infer gaze in real-time in a screen of a smartphone or tablet has been proposed. The network takes input from the patches of both eyes, the face image, and a binary mask to locate the head w.r.t. the whole image. These patches are cropped at a size of 224×244 pixels, while the face grid has size of 25×25. Conceptually, the network can be split into four sub-parts: two for the eyes (with shared weights), one for the face, and one for the face grid. The single branches are merged, after one or more FC layers, by two more layers of the same type with size, respectively, of 128 and 2. The output is the distance from the camera, expressed in centimeters.

The work in [159] infers the gaze while using two different CNNs to model the head pose and eyeball movements, respectively. Gaze prediction is extracted after aggregating the two sources of information, introducing a “gaze transform layer”. This layer encodes the transformation between the gaze vector in head and camera coordinate system without introducing any additional learnable parameter. The decomposition avoids head-gaze correlation overfitting and to re-use data designed for other tasks. Similarly, two CNNs are employed in [160], where head pose and gaze are merged and balanced at different levels. Authors incorporate different levels of representations of the head-pose into gaze estimation, namely input level, hidden feature level, model level, and task level. This way, the solution can be applied to real-world situations, i.e., in low-resolution images, in the case different lightning conditions and even if direct head-pose information is not available. Left, right eye patches, and head pose are merged with an end-to-end approach in [161], dealing with the problem of large user distances from the camera and high variations in the head pose. A motion capture system is used to find the relative pose between mobile wearable glasses and an RGB-D camera, which provides the subject head pose. Eye gaze labels are directly provided by the glasses. A network for semantic image inpaint is applied to the regions covered by the eye tracking glasses, and landmark detection network extracts the positions of five facial landmarks used to generate eye patch images. Finally, the network can be trained on the annotated gaze labels.

Another proposal to achieve end-to-end full-face gaze estimation suggests the introduction of a learnable pooling layer that removes redundant information [162]. The model can learn the size of the grid in the pooling filter, keeping the resolution of valuable regions high and compressing the input by removing redundant information, and can be learned by backpropagation. In [23], instead, spatial weights are applied on the feature map that was extracted from the facial image to obtain both 2D and 3D gaze estimation by using a CNN. The spatial weights are learned on the activation maps of the convolutional layers and they can efficiently encode the information of different regions of the image containing the full face into a standard CNN architecture. Additionally, RCNNs have been used for achieving gaze [124] combining face, eyes region, and face landmarks as individual streams. The RCNN combines appearance, shape, and temporal information, and it can leverage the temporal information of the sequence of images. For each frame, the static streams are combined in a multi-stream CNN; the feature vectors are input to a many-to-one recurrent module for predicting the gaze vector of the last sequence frame. The addition of geometry features to appearance-based methods showed a regularizing effect on the accuracy. On the other hand, a CNN to estimate the gaze using only eye patches, even in case of head rotation, has been proposed in [143]. The architecture consists of two parts performing two different regressions: the first part from an eye image to a “gazemap”, while the second from the “gazemap” to a gaze direction. The idea of this explicit prior is to regress an intermediate pictorial representation instead of directly regressing two angles that represents the pitch and yaw of the eyeball.

Instead, the two-eye asymmetry (i.e., two eyes of the same person that may appear uneven) has been taken into account with a face-based asymmetric regression-evaluation network in [163]. The regression-evaluation network (FARE-Net) includes one face-based asymmetric regression network (FAR-Net) and one evaluation network (E-Net). The first one estimates the gaze directions for both eyes asymmetrically. The second network learns, instead, the reliabilities of two eyes adjusting the regression strategy of the asymmetric mechanism. Integrating the asymmetric network with the evaluation network, the system optimizes the overall gaze estimation accuracy.

Often, uncalibrated systems still tend to obtain limited accuracy and their output usually exhibits high variance as well as subject dependent biases. An attempt to introduce subject-specific calibration images to enhance the performance of end-to-end systems has recently been proposed in [164]. The introduced approach directly trains a differential CNN to predict gaze differences of the same subject. From a set of subject-specific inferred differences, the gaze direction of a novel eye sample is predicted. This way, it is possible to provide an estimation more robust to eyelid closing or illumination perturbations. In the experiments, even one single calibration sample can outperform the performance of state-of-the-art uncalibrated solutions.

## 8. Publicly Available Datasets

In this section, major publicly datasets that are useful to benchmark gaze tracking approaches are listed. By starting this list from the task focusing on gaze estimation based on scene analysis, it is possible to assert that size of available data is the bottleneck for developing saliency models by deep neural architectures, because collecting eye-movement data is very time-consuming and expensive. Most of the current studies on human attention and saliency modeling have used high-quality stereotype stimuli. However, in the real world, captured images undergo various types of transformations.

MIT is an early dataset provided in [165], where eye tracking data of 115 viewers on 1003 images were collected and made available as train and test examples to learn models of saliency-based on low, middle, and high-level image features.

A novel saliency dataset (namely GazeGAN), including fixations of 10 observers over 1900 images degraded by 19 types of transformations, has been introduced in [166]. Together with the dataset, it is also available the implementation code (https://github.com/CZHQuality/Sal-CFS-GAN) of a new image generator allowing for common saliency nets to be trained in a more robust way against various transformations. A PCCR based eye tracker is used to record actual eye-movements.

DHF1K (Dynamic Human Fixation), another benchmark for predicting human eye movements during unconstrained dynamic viewing of the scene, has been introduced in [167]. It includes 1000 videos with more than 600,000 frames and per-frame fixation annotations from 17 observers. The sequences have been carefully collected to include diverse scenes, motion patterns, object categories, and activities. Eyes movements of the participants were monitored binocularly while using a Senso Motoric Instruments (SMI) RED 250 system (https://www.mindmetriks.com/uploads/4/4/6/0/44607631/smi_flyer_red250mobile.pdf) at a sampling rate of 250 Hz. The same authors also release the code for a video saliency model that augments the CNN-LSTM network architecture with an attention mechanism to enable fast, end-to-end saliency learning (https://github.com/wenguanwang/DHF1K).

The largest available dataset for saliency prediction is SALICON [168]: it contains 10,000 training images, 5000 validation images, and 5000 testing images, taken from the Microsoft COCO dataset. A mouse-contingent multi-resolution paradigm based on neurophysiological and psychophysical studies of peripheral vision was used to simulate the natural viewing behavior of humans. The paradigm allowed for using a general-purpose mouse instead of an eye tracker to record viewing behaviors, i.e., to build gaze ground truth data.

Finally, in order to analyze what can bias saliency in images, the dataset EMOd [169] has been introduced: it includes eye tracking data as well as extensive annotations about image contexts such as objects name and contour, together with their emotional and semantic categories. Ground truth concerning gaze directions was recorded by a PCCR based eye tracker at 1000 fps.

Concerning the egocentric view, the *Georgia Tech Egocentric Activity* datasets provide very extended benchmarks [170]. The latest version, namely EGTEA Gaze+, contains 28 h of cooking activities from 86 unique sessions of 32 subjects. These videos come with at least one gaze measurement (the 2D point on the image) of the camera wearer. Gaze measurements have been obtained using a wearable eye tracker (30 fps).

Concerning the third-person view, *GazeFollow* [95] is a dataset for benchmarking gaze following in static images. This is a large-scale dataset that is annotated with the location of where people in images are looking. Several major datasets that contain people have been used as a source of images. The dataset contains data regarding 130,339 people in 122,143 images, with annotations of the center of a person’s eyes and where the person is looking. Annotations were made by using Amazon’s Mechanical Turk (https://www.mturk.com/). The authors provide also an API (http://gazefollow.csail.mit.edu/demo.html) to return the gaze of the person and to identify the object being observed, given an image as input.

Gaze360 [171] is a large-scale gaze-tracking dataset for robust 3D gaze estimation in unconstrained images. It consists of 238 subjects in indoor and outdoor environments with labelled 3D gaze across a wide range of head poses and distances. Ground truth was built by a panoramic camera placed on a tripod in the center of the scene, and a large moving rigid target board marked with an AprilTag (https://april.eecs.umich.edu/software/apriltag).

A new video dataset for object referring, with 30,000 objects over 5000 stereo video sequences annotated via crowdsourcing for their descriptions and gaze targets has been made available into [97]. The related code for implementing a Temporal-Spatial Context Recurrent ConvNet model able to determine, by an end-to-end mode, which objects are referred (watched) by people in the scene is also available (https://people.ee.ethz.ch/˜arunv/ORGaze.html).

VideoGaze Dataset is instead a large-scale dataset for gaze-following across multiple views [103]. Annotations were made by using Amazon’s Mechanical Turk. The workers exploited an online tool appositely developed by the researchers. VideoAttentionTarget is a recently introduced dataset [98] built by gathering videos from various sources including live interviews, sitcoms, reality shows, and movie clips, all of which were available on YouTube. The annotators labeled the gaze target as a point in each frame for each annotated person in selected clips. This produced 109,574 in-frame gaze targets and 54,967 out-of-frame gaze annotations.

Similarly, VideoCoAtt, introduced in [101], is a dataset from public TV show videos, containing 380 complex video sequences with more than 492,000 frames that include diverse social scenes for shared attention study. All of the video frames have been annotated using an online tool.

Finally, it is worth naming Cityscape [172], a generic dataset that focuses on semantic understanding of urban street scenes. It has been exploited for evaluating object referring approaches.

Gaze estimation techniques effectiveness, in particular for deep learning-based methods that analyze face/eyes images of the beholder, is often dependent on the size and variability of different datasets. Many datasets have been proposed varying recording conditions, type of data, constraints, and scenarios. Many of them were initially based on high-resolution images of subjects close to the camera, with a lower level of head mobility. As long as different challenges have been partially addressed, e.g., slightly head pose variations, datasets have considered wider distances, consumer camera images, and higher head pose variability. Regarding the gaze target, discrete screen coordinates or 3D gaze directions are usually considered.

Columbia dataset [173] is composed of 5880 images of 56 people, providing variance with five different head poses and 21 gaze directions. Subjects were seated in a fixed location in front of a black background using a chin rest, and a grid of dots was attached to a wall in front of them. Five camera positions, one for each head pose, were marked on the floor at two meters from the subject. Unfocused images or cases of subject blinking have been discarded from the dataset. The images are very high in resolution, i.e., 5184×3456.

EYEDIAP [174] is composed by Microsoft Kinect and HD camera data synchronized with five LEDs of 16 people (12 males, four females) in a total of 94 sessions. Users were asked to sit in front of the setup and to watch at different targets, represented of both a 3D ball in the scene as well a circle in a discrete (i.e., appearing at random locations) or continuous (i.e., moving a random trajectory of 2 s) in the computer screen. Ground truth data of head pose and eye tracking are generated by an algorithm, enriched with manually annotated data for unreliable images (e.g., when the user blinks), but only for a subset of sequences.

UT Multiview dataset [126] is composed of video sequences of 50 participants (35 males, 15 females) from eight different views for a total of 160,000 images. Users are located at 60 cm from the screen using a chin rest and are asked to look at a circle appearing in random locations among 160 different discrete cells. 2D and 3D facial landmarks annotations together with ground truth gaze locations are provided.

MPIIGaze dataset [157] contains 213,659 images collected from 15 participants during natural everyday laptop use over more than three months. Images have been acquired from the onboard camera. The dataset is significantly variable with respect to appearance and illumination.

TabletGaze dataset [150] consists of 51 subjects, each with four different postures and 35 gaze locations. Subjects vary in the appearance, e.g., race, gender, presence of glasses. The faces of the subjects were acquired from the onboard camera of a tablet. During the data collection process, the subject motion was not restricted, and each subject performed four body postures: standing, sitting, slouching, and lying.

RT-GENE dataset [161] deals with large camera-to-subject distances and high variations in head pose and eye gaze angles. In fact, in datasets where subjects are directly facing a screen or mobile device, the distance between subject and camera is relatively small, as well as head pose variations. On the contrary, datasets that take into account wider distance present only head pose information (and we excluded them from this section). Thus, RGBD data of a subject wearing a mobile eye tracker are recorded. To avoid changes in the user appearance due to the presence of glasses, the authors used semantic inpainting [175,176] of the regions covered by the eye tracking glasses.

The first large-scale dataset is GazeCapture (http://gazecapture.csail.mit.edu) [158], composed of 1450 people with a wide variety of backgrounds and lighting conditions with unconstrained head motion. Authors also deal with the problem of reliability and variability in the case of participants not taken from a laboratory environment. They used crowdfunding and asked the user to change the orientation of the mobile every 60 s to encourage variability, and proposed to force the airplane mode to be off to avoid user distractions, showing a pulsating dot for approximately 2 s before starting the recording (0.5 s).

Finally, it is worth to mention the category of synthetic datasets that arbitrarily simulate head pose variations and user distances, getting annotated ground truth data in any circumstance. SynthesEyes [177] and UnityEyes [178] are two examples of eye-patch synthetically generated and used to infer the gaze. In the first one, authors rendered a generic dataset of 11,382 images covering 40${}^{\circ }$ head pose variation and 90${}^{\circ }$ of gaze variation. Eye color and environmental lighting are randomly sampled for each image. The latter is much bigger thanks to improved performance of the rendering during the generation process. Authors also released a tool to generate customized data and setting own camera and eye parameters. However, it is still challenging to apply a network trained on synthetic data on real images, although this consideration is valid for deep learning in general, representing a very hot research topic [179,180].

A summary of the aforementioned datasets is reported in Table 1. For each entry, the following data are reported: the number of people, the acceptable head pose variations, the ranges of user distance from the sensor(s), the number of discrete gaze targets (or if they are continuous), the number of images (or the temporal length if data are in the form of videos), the data type, and their spatial resolution.

## 9. Metrics and Evaluation Criteria

In this section, the most common metrics reported in the state of the art to measure the performance of each proposed approach are reported and discussed.

Concerning gaze prediction from saliency, a large variety of metrics exists [181]. They can be mainly categorized in location-based and distribution-based metrics. The first category considers saliency maps at discrete fixation locations, while the second treats both ground-truth fixation maps and predicted saliency maps as continuous distributions. When looking at saliency as a gaze predictor, it is more suitable to consider discrete fixations; in this way, the problem becomes closer to that one posed by the other approaches that are differently categorized in this manuscript, making an overall evaluation possible.

Under this premise, suitable metrics are the Area Under Curve (AUC), the Normalized Scanpath Saliency (NSS), and the Information Gain (IG).

Normalized Scanpath Saliency (NSS) is a simple correspondence measure computed at fixed locations between the estimated saliency map and the ground truth, performed after a normalization task in the saliency scores in order to have zero mean and unit standard deviation. NSS is computed once for each saccade, and, subsequently, the mean and standard error are computed across the set of NSS scores. NSS=1 indicates that the subjects’ eye positions fall in a region whose predicted density is one standard deviation above the average. In contrast, NSS=0 indicates that the model performs no better than picking a random position on the map. NSS is sensitive to false positives, relative differences in saliency across the image, and general monotonic transformations. However, because the mean saliency value is subtracted during computation, NSS is invariant to linear transformations.

Area Under Curve (AUC) is the area under Receiver Operating Characteristic (ROC) i.e., the curve of true positive rate versus false positive rate for different thresholds on the predicted gaze map. Using this measure, the saliency map is treated as a binary classifier on every pixel in the image; pixels with larger saliency values than a threshold are classified as fixated while the rest of the pixels are classified as non-fixated. Human fixations are used as ground truth. By varying the threshold, the ROC curve is drawn as the false positive versus true positive rate, and the area under this curve indicates how well the saliency map predicts actual human eye fixations. Perfect prediction corresponds to a score of one. Different AUC implementations differ in how true and false positives are calculated. At least two variants deserve mention i.e., AUC-Judd (AUC-j) and AUC-Borji (AUC-b), which are quite commonly exploited for evaluating the estimates of fixations as a pure classification problem. Finally, a variant that penalizes models for center bias (i.e., the natural tendency of observers to preferentially look towards the center of images) is the so-called shuffled AUC (sAUC).

Information Gain (IG) is an information-theoretic metric that measures saliency model performance beyond systematic biases. It assumes that the input saliency maps are probabilistic and it requires careful modeling of these biases.

AUC is exploited in gaze estimation from egocentric (first-person) and third-person views.

Another common metric is the Average Angular Error (AAE) i.e., the average angular distance between the predicted and the ground truth gaze positions. In the object referring problem, sometimes a different metric is used. It is named Acc@1 and it refers to the percentage of top-scoring candidates being a true target gaze detection. A candidate is regarded as a true detection if the Intersection over Union (IoU) computed between the predicted bounding box and ground truth box is more than 0.5. Finally, also the $L^{2}$ distance, measured in pixel and computed between the predicted shared attention box and the annotated ground truth, is sometimes used.

Face-eye analysis based gaze estimation is evaluated w.r.t. the precision of the estimated PoR against the ground truth information. A common metric is the mean error, which is often provided together with the standard deviation, and usually measured in degrees or in centimeters. When the 3D gaze vector is evaluated, the AAE mentioned above is used. Additionally, the average Euclidean distance from the location of the true fixation is a very common metric. In most of the cases, evaluation is given by computing metrics on a specific subset of images. Differences are computed for each sample of the test set and summed up using mean absolute error or root square mean error. To take into account the case of a video stream, the dot error is introduced. It is defined as the average prediction of all the consecutive frames corresponding to the same gaze fixation. Depending on whether the proposed method is using one, two, or both eyes, the metrics are subsequently computed for one or two eyes, also w.r.t. the information available in the employed dataset. Finally, if a gaze hit is evaluated in a set of possible discrete positions, the problem is modeled as a classification; thus, commonly employed metrics are employed, mainly precision and accuracy. Finally, for the case of yaw and pitch angles computed separately, the aforementioned metrics are axis-based computed.

The above metrics are related to the accuracy but, of course, it is not the only factor to be taken into account when evaluating a gaze estimator.

In light of this, it is possible to also use the following non-functional requirements and criteria to classify different gaze tracking methods:

- technology requirements;
- grade of invasiveness;
- speed/computational load;
- the need (and its level) of human intervention; and,
- the availability of open source code/data. (reproducibility).

Concerning technology requirements, gaze tracking solutions can use single or multi-camera solutions, but also eyeglasses or head-mounted display. This has clear implications on the grade of invasiveness for the user.

Multi-camera systems exploit the well-known a priori geometry of multiple views while achieving a gaze tracker with a single camera is a more challenging task. Excluding specific ad-hoc hardware configurations, which represent the highest grade of invasiveness, camera-based systems gaze tracking methods can employ RGB cameras or depth sensors (RGBD sensors). The latter family of devices provides both RGB and depth channels thanks to whom, for each 2D pixel, its physical distance from the sensor origin is given. Anyway, using a single camera can remove many constraints, representing the least invasive solution, in particular, as compared with head-mounted devices; furthermore, it is also a cheaper solution.

Regarding the calibration phase, especially in the last few years, user-independent systems have been introduced, with the advantage of being more portable and usable in all of the situations where a calibration procedure is not possible, like in the case of market analysis and disabilities. Of course, this generally comes with the price of a precision loss.

A very relevant aspect, which is often given little academic importance, is the capability of the methods to work in real-time, a fundamental requirement in most of the practical and commercial scenarios. There is an objective difficulty in the definition of real-time gaze estimation since it does not exist a fixed minimum frame rate to be achieved since it can vary with the particular application context. Besides, deep learning based approaches can only run in real-time on expensive GPUs, and then they should be evaluated in terms of cost and portability.

Finally, for an exhaustive evaluation, the possibility of getting code and datasets related to the available solutions in order to personalize or improve the provided model should be taken into account. Datasets represent the fundamental feature to ensure reproducibility, and they have been already introduced and discussed in Section 8. Regarding available implementations, it is worth noting that early gaze estimation works usually did not release any resource. Instead, this practice has become more common among years, and definitely spread with deep learning framework like PyTorch (https://pytorch.org/) and TensorFlow (https://www.tensorflow.org/), as well as free and open online resources, like Papers With Code (https://paperswithcode.com/). The latter mainly focuses on machine learning papers, providing code and evaluation tables, with official or unofficial implementation.

When considering all of the concepts introduced herein, a scheme summing up the principal methods reviewed in this document is reported in Table 2. Note that every categorization becomes necessarily reductive. For instance, the error cannot be evaluated in an absolute way, since it must be related to the experimental setup (especially in terms of the user distance from the sensor and of the employed dataset to validate the method); similarly, other features and the innovation introduced itself cannot be evaluated singularly, and must be related to the scope and scenarios of each paper.

Regarding the error for the beholder face case, the error is reported in degrees when possible, considering the AAE as a metric. It is easy to note how the error analysis is much more valid and can only create a real comparison in the case of shared datasets used for the experimental evaluation. Anyway, it is also important to note that different authors can often opt for different types of validation (e.g., different values of *k* in the k-fold validation). For such a level of detail, the reader should not only consider the single metric, but a comprehensive evaluation by consulting the original manuscript is recommended.

From the metrics in the table, it is possible to derive the importance of having shared benchmarks. Before their introduction, in some cases, the methods reported excellent results even better than those that were achieved in the last years by exploiting complex deep learning architectures. It is possible to guess that this oddity is due to the fact that former analysis was done on limited and not challenging datasets and especially through algorithms built well-knowing the data on which they had to work.

However, we also think that it is very useful to provide an overall view at a glance, reporting, in particular, the main datasets used for the evaluation, as well as illustrating the reproducibility of the results. Regarding the latter, we consider the results to be reproducible if publicly available datasets have been employed and if source code is provided. We apologize in advance with those authors having a code implementation available online, but not mentioned in this manuscript. This could have happened when the repository is not directly attributable to one of the authors, or an unofficial but faithful implementation exists.

The best results have been reported in the case of single work that proposes multiple architectures with major or big variations. Anyway, it is important to note that this has an important implication, i.e., that, if the theoretical result is satisfactory, it is often not possible to automatically understand which of the possible variations is more suitable in an uncontrolled environment. This is consistent with the well-known gap between theoretical results and their applicability, which is also reflected in the gaze estimation problem.

If compared with appearance-based methods, the accuracy of geometric methods is generally lower; moreover, it is unclear whether shape-based approaches can robustly handle low image quality. While appearance-based models create a mapping scheme between the input image and the observed point, geometric models track the gaze by intersecting the straight line with the environment. In both cases, the final outcome is the PoR that can be on the user screen or a 3D point of a known environment. Having no feature detection step, the appearance-based method is more suited to handle lower resolution inputs. Initially, all of the methods that were based on the observation of the beholder eye/face were too inferior to ad-hoc eye tracker in terms of performance, in terms of overall accuracy, but especially in the case of head pose variations, getting mainly purely academic interest. However, the evolution of the hardware and the advancements of the whole computer vision field noticeably reduced this gap, allowing for the use of computer vision trackers in real scenarios [182]. Regarding hardware, computer-vision based gaze estimation, at first, required specialized expensive hardware or illuminators. With the availability of sensor-rich and powerful devices, the premises to obtain pervasive gaze tracking with off-the-shelf sensors have been fulfilled [183]. Recently, the availability of GPUs and other technological advancements have boosted the process of making end-to-end approaches that are indisputably the most promising and investigated approach for achieving gaze estimation from digital visible-light images.

It is straightforward to observe that in the first-person view the accuracy is very high even if no prior information about the relationships between acquisition device and the environment is known. The beholder can freely move and no calibration can be made, but, despite these relaxations, outcomes can be compared with those of methods leveraging eye/face analysis. This is mainly due to the possibility to analyze the scene from the eye of the beholder that allows for exploiting his interaction with the framed objects. This brings about another relevant consideration: in this research area, temporal analysis plays a central role to get good performance. This is also now well established, because modern neural networks allow for modeling the temporal variations very well by analyzing entire sequences of data. Data modeling is also supported by the availability of precise ground truth data generally gathered by PCCR based eye trackers. Of course, in the case of unsupervised approaches for segmenting sequences, i.e., spatial and temporal features, the accuracy decreases. First-person view approaches require wearable devices and they are not suitable in the case of ecological analysis. They cannot be made transparent to the beholder and they then ineluctably introduce a bias in the scene exploration. This could raise an issue, in particular application contexts e.g., assistive technologies for the diagnosis of assessment of cognitive diseases. Moreover, most of the methods leveraging the first-person view requiring the user to avoid quick head movements, to acquire also his hands while manipulating objects, and to operate in not very cluttered environments, which frequently led to object misclassification and/or wrong keypoints matching that are very common methodological fundaments.

Quite similar considerations can be made for approaches that rely on visual saliency, but gaze track predictions may be less accurate than the other cases since they are only completely based on scene contents. The scene is, in fact, acquired from a different point of view and this introduces an additional uncertainty (for instance, the center bias) that the models have to handle. This is done, for example, by different loss functions (allowing multiple learned priors), performing an additional cross-disciplinary evaluation on elicitation stimuli, adding depth information, even if displayed on a 2D screen or at least, by combining information coming from a camera looking at the user’s face. In light of this, saliency-based gaze estimators are better suited to work in conjunction with other approaches, allowing them to avoid personal calibration or increase precision. Anyway, they could be stand-alone used when the gaze is a marginal characteristic to be monitored in a person’s behavior and, therefore, a rough estimate may be enough without using devices dedicated to the acquisition of the observer.

Gaze estimation in third-person views, and even more the shared attention prediction, are also very challenging tasks, because, in these cases, affordable ground truth data are not easy to be collected. Differently for the other categories, PCCR based eye trackers cannot be used, thus ground truth data are generally collected by manual annotations through crowdsourcing. This makes harder the direct comparison with other categories of methods. Anyway, also in this area, improvements that are achieved by deep learning are evident, so that even joint and shared attention tasks from third-person views have been recently faced. Anyway reached accuracy is still far from reaching human performance. The presence of several salient objects in the scene, several people looking at each other, one or more people interacting with one or more objects, the head pose that cannot be well estimated due to occlusions or that cannot be accurately perceived for example for inappropriate point of view are common situations to be addressed in real contexts but still not fixed by the proposed approaches. A key still unsatisfied requirement that could help to overcome these drawbacks is the availability of huge annotated datasets tailored for this problem in order to train models based on a multi-task learning framework. A different way to get around the aforementioned problems is to exploit temporal cues (i.e., using videos instead of a static image) and/or additional prior information like language (object referring problem) or multiple persons looking at the same target (shared/joint attention).

## 10. Discussion

This section discusses the reported methodological progress and tries to identify what are the possible future scenarios also in consideration of the most recent research lines in the computer vision and machine learning field. Analyzing at the works reported in this survey, it is possible to conclude that we are far from having the definitive gaze tracker and that each method will present a necessary trade-off between possible advantages and disadvantages. From one side, thanks to the availability of larger datasets, but also when considering the boost achieved with deep learning, not only in terms of new architectures but also in terms of code reusability and high-level frameworks (e.g., PyTorch, TensorFlow, Keras), it is possible to observe how reproducibility has become more frequent than the past few years. Consequently, many of the works that have been introduced in Section 7.2, especially for the case of end-to-end systems (Section 7.2.2), present experimental evaluation on one or more datasets, with a frequently available implementation.

Additionally, it emerges how it is very difficult to evaluate methods all together using a single metric. The presence of constraints, calibration, or pre-processing step as well as the intrinsic characteristics of the datasets could make a performing method very difficult to be applied in unconstrained scenarios. In other cases, in the same work, many slightly different architectures or sets of parameters are introduced, but the best performance is only achieved when different combinations are considered during different experimental phases: in uncontrolled scenarios, it would be difficult to automatically understand the precise configuration to apply.

Anyway, it is worth noting that the evaluation of gaze estimation solutions depends on its application scope. For example, tracking the focus of attention using only head pose information could be a suitable solution for some daily-life scenarios, while, for clinical scopes, very high precision is required and no one of the works in the literature could be still adequate. Analogously, there is a trade-off choice between precision and computational time that has to be made when considering the final application aim. If offline processing can be carried out then the highest quality of input images and complex architectures are allowed, whereas, if real-time feedbacks are required, then lighter models and coarser data might represent the best setup. Another relevant aspect is the invasiveness (in terms of human intervention e.g., personalized calibration) and intrusiveness (e.g., how much the technology is perceived by the end-user i.e., his technology awareness) of the solutions for gaze tracking. For example, a clinical study that involves people with a neurological disability, like autism spectrum disorders, should use uncalibrated and non-intrusive solutions, since people could be reluctant to invasive devices or unable to perform calibration procedures. Conversely, laboratory test performed with collaborative users can employ a long calibration procedure and invasive hardware, and so on. In light of this, we think that the proposed taxonomy can help in identifying the adequate method, depending on the priorities and application context under consideration.

To help the reader to navigate in this broad class of methods in the practice, Table 3 provides insights on how the aforementioned attributes affect the working conditions of methods. The main aim is trying to mix both the research and application perspectives and highlight their different needs. In particular, an attempt to directly associate the application requirements with the schematic representation in Table 2 has been provided. For example, for HRI studies in real and complex scenarios, speed would represent a critical issue: in fact, getting quick estimations of the user gaze is the only mean to register parameters, like the user engagement, or to achieve the proper user awareness, and to safely take the proper consequent decisions/actions [184,185,186]; as a consequence, real-time gaze estimation systems are more likely to be preferred [161,187,188]. From the other side, even in the same application domain, it can happen that different attributes could be prioritized, depending on the specific instance of the problem. For example, a market analysis application in public spaces and retrieving data from crowds are likely to require the least possible intrusiveness level at the price of getting rough estimations [38]. However, the same goal could be reached through recruited volunteers who may be willing to use intrusive devices and time-consuming calibration phases, this way achieving the maximum possible precision [75,76]. Anyway, once the priorities in terms of attributes have been fixed, we hope that a similar work, as done in Table 2, can help the reader to select the most suitable (family of) methods.

In this last part of this section, a glimpse of possible future research directions is given. Starting from the qualitative and quantitative evaluations reported in Section 9, it is possible to derive that the way to improve gaze tracking is to use more efficient underlying algorithms such as those for recognizing objects (in the case of methods based on scene analysis), which could allow for better generalization and therefore optimal operation even in scenes cluttered or in the presence of distortions and noise in the images. From this perspective, of course, deep learning has represented a breakthrough, allowing researchers to achieve better gaze tracking performance. It has also changed the way of dealing with the problems by introducing the possibility to take multiple cues in the spatial and temporal domains into account. Anyway, a very relevant issue has been emerging: the proposed gaze tracking approaches appear too much constrained to the domain in what they are conceived. Learning strategies, data collected, and underlying ideas may be less effective if moved into another application domain. This means that the knowledge generalization on unseen testing data, which may have a different distribution of that of the training set, is not trivial. For example, the models of saliency learned in a street scene are not applicable in an indoor scenario, and then new models have to designed and learned to accomplish a domain change. Another important aspect regarding learning architectures, which, in almost all cases, exploit well-known models (backbones), ready to use and widely used for the solution of basic tasks (e.g., object recognition). Besides, related parameters are inherited from other application sectors and just fine-tuned for specific purposes. Finally, another problem concerns the availability of accurately annotated data is a key aspect of well-established deep learning methods. Anyway, some recent discoveries in computer vision and machine learning can fix the aforementioned issues and then the research outcomes should be taken and adapted for gaze tracking purposes. For example, it could be interesting to exploit more modern architectures that explicitly learn spatial transformations and temporal feature alignment without leading to high-dimensional feature maps [189,190]. This could improve object recognition and scene analysis can benefit from improving gaze tracking from first and third-person view, and saliency estimation as well. Moreover, upcoming research could resort unsupervised domain adaptation methods which address the domain-shift problem without using ground truth labels in the target domain [191]. For example, meta-learning [192] is capable of fast adaptation to new camera scenes and it is compatible with any model trained with gradient descent and applicable to a variety of different learning problems, including classification, regression, and reinforcement learning. Another approach that could be exploited is the so-called self-supervised learning, in which knowledge can be transferred by reducing a learned representation to pseudo-labels on an unlabeled dataset [193]. Differently, few-shot learning techniques could be considered to learn a model from limited training examples for a task [194]. Finally, incremental learning [195], i.e., a method of machine learning in which input data are continuously used to extend the existing model’s knowledge, could also be taken into consideration to push up the flexibility and usability of computer vision-based gaze trackers. This could solve the critical issue of lack of annotated data or, at least of precisely annotated data when this is achieved by manual annotation by crowdsourcing.

## 11. Conclusions

In this work, a detailed analysis of the literature has been given, discussing how the great advancements in computer vision and machine learning have impacted gaze tracking in the last decade. Besides, a wider viewpoint on this topic has been proposed that has brought to a new taxonomy by considering gaze tracking as a more exhaustive task that aims to estimate gaze target from different perspectives: from the eye of the beholder (first-person view), from an external camera framing the beholder’s, from a third-person view looking at the scene where the beholder is placed in, and from an external view independent from the beholder. From this analysis has emerged that computer vision allowed for gaze tracking systems to become increasingly accurate, even if it is very difficult to evaluate methods all together using a single metric. Moreover, it has been pointed out that most of the approaches were validated on large datasets whose characteristics could make it very difficult to move a method greatly performing in a scenario to others, eventually fully unconstrained, ones. Another emerged issue concerns that, sometimes, in the same work, slightly different architectures or configurations of parameters are introduced, and it would be difficult to automatically understand the precise configuration to apply in new scenarios. On the other hand, the availability of larger datasets and the boost achieved with deep learning, also in terms of code reusability and high-level frameworks, made reproducibility more common than in the past. As a general conclusion, it is then possible to state that the evaluation of a single gaze estimation solution is ineluctably related to its application range and scope and, thus, it is definitely possible to assert that there is no “killer app” yet. All of the proposed approaches have both pros and cons and then the choice of the most suitable one has to be made time to time taking many factors into account. The last part of the document gave also a glimpse of possible pathways to overcoming the limitations by exploiting recent deep learning architectures, not extensively exploited for gaze tracking yet.

## Figures and Tables

**Table 1:** A summary of relevant datasets for gaze estimation. *cont.* stands for continuous, *E/F* for eye/face, *pos.* for position, *coord.* for coordinates, *img.* for images.

Dataset	Taxonomy	Type of Content	# People	# Head Poses	User Distance	Gaze Target	# Images	Data Type	Resolution
Columbia [173]	Beholder E/F	facial img. (lab setup)	56	5	2 m	21	5880	color	5184×3456
EYEDIAP [174]	Beholder E/F	facial img. (lab setup)	16	cont.	0.8–1.2 m	cont.	videos (>4 h)	color+depth	1920×1080 (camera) 640×480 (Kinect)
UT Multiview [126]	Beholder E/F	eye region img. + synthetic 3D model (lab setup)	50	8	0.6 m	160	64,000	color	1280×1024 (eye crop provided)
MPIIGaze [157]	Beholder E/F	facial img. (consumer camera)	15	cont.	0.4–0.6 m	cont.	213,659	color	1280×720 (eye crop provided)
TabletGaze [150]	Beholder E/F	facial img. (consumer camera)	51	cont.	0.3–0.5 m	35	videos (∼24 h)	color	1280×720
GazeCapture [158]	Beholder E/F	facial img. (consumer camera)	1474	cont.	very close	cont.	2,445,504	color	1000×1000
RT-GENE [161]	Beholder E/F	facial + wereable device img. (lab setup)	15	cont.	0.5–2.9 m	cont.	122,531	color + depth	1920×1080 (color) 512×424 (depth)
SynthesEyes [177]	Beholder E/F	synthetic eye patches	n.a.	n.a.	adjustable	cont.	11,382	color	120×80
UnitEyes [178]	Beholder E/F	synthetic eye patches	n.a.	n.a.	adjustable	cont.	1,000,000	color	400×300
GazeGAN [166]	Scene Saliency	scene + fixations	10	free-viewing	0.6 m	cont. (24” display)	1900	color	1920×1080
DHF1K [167]	Scene Saliency	scene + fixations	17	headrest constrained	0.68 m	cont. (19” display)	1 k videos (600 k frames)	color	640×360
SALICON [168]	Scene Saliency	scene + visual attention	16	free-viewing	variable	cont.	10,000 images	color	640×480
EMOd [169]	Scene Saliency	scene + eye movements + emotions + objects + semantics	16	free-viewing	variable	cont. (22”display)	1019	color	1920×1080
EGTEA Gaze+ [170]	Egocentric Vision	scene + 2D gaze point on the frame	32	free-viewing	variable	cont.	videos (29 h)	color	640×480
GazeFollow [95]	Third-person view	scene + head pos. + target pos.	130,339	free-viewing	variable	cont.	122, 143 images	color	var.
Gaze360 [171]	Third-person view	subject + target + 3D gaze	238	free-viewing	variable	cont.	129 K training, 17 K validation and 26 K test	color	3382×4096
Object Referring [97]	Third-person view	object bounding box + language description + gazes	20	free-viewing	variable	cont.	5000 stereo videos	color	1600×1200
VideoGaze [103]	Third-person view	scene + target + gaze annotations	47,456	free-viewing	variable	cont.	140 movies	color	var.
VideoCoAtt [101]	Third-person view	heads + gaze directions	n.a.	free-viewing	variable	cont.	380 videos (492,100 frames)	color	480×320

**Table 2:** A schematic representation of the most important works cited in this document. For each entry, we sum up the taxonomy and eventual technical requirements, as well as other constraints, and performance. The ain datasets whose authors used in the experimental phase are reported. If no dataset is reported, experiments have been done on data not make publicly available or proprietary. The reproducibility of the method in the table means that the code has been made available by the authors and presented results have been gathered on publicly available data; *calib.* stands for calibration, *h.p.* for head pose, *DL* for deep learning.

Method	Taxonomy	Tech Req	Speed/Load	Constraints	Reproducibility	Metric	Notes
[144] (2012)	Feature	Head-mounted		person calib.		$1.40^{\circ }$ (with 100 training samples)	
[139] (2014)	Feature	RGB Camera	28 fps	person calib., fixed h.p.		$0.97^{\circ }$ (with 9 training samples)	
[150] (2017)	Feature	RGB Camera				3.63–6.03${}^{\circ }$ person indep.	
						2.86–4.76${}^{\circ }$ person dep.	
[128] (2008)	Geometric	RGB Camera		person calib.		2.18 ${\left(x\right)}^{\circ }$–2.53${\left(y\right)}^{\circ }$	
[36] (2015)	Geometric	RGBD Camera	12 fps	person calib.		1.38–2.71${}^{\circ }$	
[73] (2016)	Geometric	RGBD Camera	8.66 fps			$2.48^{\circ }$	
[136] (2017)	Geometric	RGBD Camera	-	person calib. (1 point)		$1.99^{\circ }$	
[158] (2016)	End-to-end	RGB Camera	10–15 fps		✓	2.58 cm (iTracker)	analysis of calib./non-calib. cases
						3.63 cm (MPIIGaze)	
						3.17 cm (TabletGaze)	
[157] (2015)	End-to-end	RGB Camera			✓	6.3${}^{\circ }$ (MPIIGaze)	
[159] (2017)	End-to-end	RGB Camera	1000 fps			5.6${}^{\circ }$ (small network)	face alignment time
						$4.3^{\circ }$ (deep network)	to be considered
[23] (2017)	End-to-end	RGB Camera			✓	<5${}^{\circ }$ (MPIIGaze)	also 2D error analysis
						<$6^{\circ }$ (EYEDIAP)	computed in the manuscript
[143] (2018)	End-to-end	RGB Camera			✓	$4.5^{\circ }$ (MPIIGaze)	
						$10.3^{\circ }$ (EYEDIAP)	
						$3.8^{\circ }$ (COLUMBIA)	
[161] (2018)	End-to-end	RGB Camera	25.3 fps		✓	$4.3^{\circ }$ (MPIIGaze)	results with inpainting
						$7.7^{\circ }$ (RT-GENE)	during the training
[164] (2019)	End-to-end	RGB Camera	666 fps	person calib.		$3.0^{\circ }$ (EYEDIAP)	analysis on eye patch
						$3.80^{\circ }$ (MPIIGaze)	
						$3.77^{\circ }$ (UT Multiview)	
[163] (2020)	End-to-end	RGB Camera				$4.3^{\circ }$ (MPIIGaze)	
						$5.71^{\circ }$ (EYEDIAP)	
						$8.4^{\circ }$ (RT-GENE)	
[151] (2016)	Feature	RGBD Camera				$1.7^{\circ }$ (EYEDIAP, static pose, person dep.)	complete invariance analysis
						$6^{\circ }$ (EYEDIAP, invariant)	in the manuscript
[124] (2018)	End-to-end	RGB Camera			✓	3.4${}^{\circ }$ (EYEDIAP, computer screen)	temporal model
						$5.19^{\circ }$ (EYEDIAP, floating target)	
[74] (2013)	First-person	Glasses				AUC 86.7% (GTEA Gaze+)	temporal dynamics
						AAE $7.93^{\circ }$ (GTEA Gaze+ )		
[75] (2018)	First-person	Glasses			✓	AUC 95.7% (GTEA Gaze +)	DL for temporal dynamics and	
						AAE $4.00^{\circ }$ (GTEA Gaze +)	image regions
[76] (2019)	First-person	Glasses	13–18 fps			AAE $6.2^{\circ }$ (GTEA Gaze +)	gaze as a classification and	
							regression problem
[82] (2020)	First-person	Glasses				AAE $12.3^{\circ }$ (GTEA Gaze +)	unsupervised modelling of	
							spatial-temporal features
[85] (2010)	Visual Saliency	RGB Scene				AUC 90% (proprierary)	empirical parameter settings	
[86] (2019)	Visual Saliency	RGB (Scene + Camera)		fixed h.p. and positions		AUC 60.82% (MIT)	top-down and bottom-up maps	
[89] (2017)	Visual Saliency	RGBD Scene				AUC 75%	depth, color, motion are learned
[90] (2019)	Visual Saliency	RGB Scene				sAUC 83% (EMOd)		
						AUC-j 78% (EMOd)	
						NSS 1.85 (EMOd)	
						IG 1.66 (EMOd)	cross disciplinary priors
[91] (2018)	Visual Saliency	RGB Scene			✓	AUC 88.3% (SALICON test set)	multiple learned
						NSS 3.20 (SALICON)	priors and features
[93] (2019)	Visual Saliency	RGB Scene				3.8 cm	adaptable to any CNN model
[94] (2013)	Third-person		10 fps	only h.p.		<0.5 rad${}^{2}$ MSE	realistic settings
[95] (2016)	Third-person	RGB Scene			✓	AUC 0.878 (GazeFollow)	single picture
[96] (2018)	Third-person	RGB Scene				AUC 0.896 (GazeFollow)	cross-domain
						$6.4^{\circ }$ (EYEDIAP)	handle gaze out of frame
[97] (2018)	Third-person	RGB Scene				(mean)Acc@1 47.012 (Cityscape)	object referring
[98] (2020)	Third-person	RGB Scene			✓	$L^{2}$83.3 pixels (VideoCoAtt)	shared attention
							time-varying attention targets
[99] (2020)	Third-person	RGB Scene				$L^{2}$62.84 pixels (VideoCoAtt)	end-to-end
							joint attention videos
[103] (2016)	Third-person	RGB (Scene + Camera)				AUC 84.4 (VideoGaze)	gaze across views

**Table 3:** A summary of the main attributes of a gaze estimation system, putting in evidence advantages and disadvantages of each category.

Attribute	Category	Advantages	Disadvantages
Intrusiveness	Intrusive	Most precise methods in the state of the art	Difficult to use with reluctant people Not suited for market analysis Can distort/bias the experiment
	Non-intrusive	Usable with reluctant people Usable in every scenario User not biased from sensor presence	Cannot track all of the eye features More imprecise
Number of sensors	Single	Cheaper	Less precision 3D only estimable
	Multi	3D reconstruction possible More precise	More expensive
Type of Camera	RGB	More usability	Less precision 3D only estimable
	RGBD	3D data available	More expensive
User Calibration	Calibrated	More precise	Difficult to use with reluctant people
	User Independent	Portable	Less precise
Speed	Real-time	Most of real applications require real-time	Less precision
	Near real-time/Offline	More precision	Can be employed only where time is not an issue.

## Abbreviations

The following abbreviations are used in this manuscript:

HCI	Human-Computer Interaction
HRI	Human-Robot Interaction
CNN	Convolutional Neural Network
LSTM	Long Short-Term Memory
RCNN	Recurrent Convolutional Neural Network
PoR	Point of Regard
RF	Random Forest
PCCR	Pupil Center Corneal Reflection
SVR	Support Vector Regression
mHoG	multi-level Histogram of oriented Gradients
AUC	Area Under Curve
NSS	Normalized Scanpath Saliency
ROC	Receiver Operating Characteristic
AAE	Average Angular Error
IG	information gain
FC	fully-connected
EC-IC	eye corner-iris centre
